# Lymphostatin, a virulence factor of attaching and effacing *Escherichia coli*, inhibits proliferation and cytokine responses of human T cells in a manner associated with cell cycle arrest but not apoptosis or necrosis

**DOI:** 10.3389/fcimb.2022.941939

**Published:** 2022-07-29

**Authors:** Nattaya Ruamsap, Donporn Riyapa, Sujintana Janesomboon, Joanne M. Stevens, Sathit Pichyangkul, Kovit Pattanapanyasat, Samandra T. Demons, Mark P. Stevens, Sunee Korbsrisate

**Affiliations:** ^1^Department of Immunology, Faculty of Medicine Siriraj Hospital, Mahidol University, Bangkok, Thailand; ^2^Department of Bacterial and Parasitic Diseases, Armed Forces Research Institute of Medical Sciences, Bangkok, Thailand; ^3^Center for Research and Innovation, Faculty of Medical Technology, Mahidol University, Nakhon Pathom, Thailand; ^4^The Roslin Institute and Royal (Dick) School of Veterinary Studies, University of Edinburgh, Easter Bush, Edinburgh, United Kingdom; ^5^Department for Research and Development, Siriraj Center of Research Excellence for Microparticle and Exosome in Diseases, Faculty of Medicine Siriraj Hospital, Mahidol University, Bangkok, Thailand

**Keywords:** lymphostatin, enteropathogenic Escherichia coli, inhibit T cell proliferation, cytokine suppression, G0/G1 cell cycle arrest, cyclin expression, apoptosis and necrosis

## Abstract

Lymphostatin is a virulence factor of enteropathogenic *E. coli* (EPEC) and non-O157 serogroup enterohaemorrhagic *E. coli*. Previous studies using whole-cell lysates of EPEC showed that lymphostatin inhibits the mitogen-activated proliferation of bulk human peripheral blood mononuclear cells (PBMCs) and the production of cytokines IL-2, IL-4, IL-5, and IFN-γ. Here, we used highly purified lymphostatin and PBMC-derived T cells to show that lymphostatin inhibits anti-CD3/anti-CD28-activated proliferation of human CD4^+^ and CD8^+^ T cells and blocks the synthesis of IL-2, IL-4, IL-10 and IFN-γ without affecting cell viability and in a manner dependent on an N-terminal DTD glycosyltransferase motif. Such inhibition was not observed with T cells activated by phorbol 12-myristate 13-acetate and ionomycin, implying that lymphostatin targets T cell receptor signaling. Analysis of the expression of CD69 indicated that lymphostatin suppresses T cell activation at an early stage and no impacts on apoptosis or necrosis were observed. Flow cytometric analysis of the DNA content of lymphostatin-treated CD4^+^ and CD8^+^ T cells showed a concentration- and DTD-dependent accumulation of the cells in the G0/G1 phase of the cell cycle, and corresponding reduction of the percentage of cells in S phase. Consistent with this, we found a marked reduction in the abundance of cyclins D3, E and A and loss of phosphorylated Rb over time in activated T cells from 8 donors treated with lymphostatin. Moreover, the cyclin-dependent kinase (cdk) inhibitor p27^kip1^, which inhibits progression of the cell cycle at G1 by acting on cyclin E-cdk2 or cyclin D-cdk4 complexes, was found to be accumulated in lymphostatin-treated T cells. Analysis of the abundance of phosphorylated kinases involved in signal transduction found that 30 of 39 were reduced in abundance following lymphostatin treatment of T cells from 5 donors, albeit not significantly so. Our data provide novel insights into the mode of action of lymphostatin on human T lymphocytes.

## Introduction

Attaching and effacing *Escherichia coli* are diarrhoeal pathogens of global importance. They belong to one of two pathotypes; enteropathogenic *E. coli* (EPEC) and enterohaemorrhagic *E. coli* (EHEC), the latter being distinguished by their ability to produce one or more Shiga toxins. EPEC is highly associated with infantile diarrhea in the low-income countries (Hartland and Leong, 2013) and it is common cause of traveler’s diarrhea (Laaveri et al., 2018), while EHEC remains high prevalence in industrialized countries (Hartland and Leong, 2013). Klapproth and colleagues (Klapproth et al., 1995; Klapproth et al., 1996) reported that EPEC lysates are capable of inhibiting the mitogen-activated proliferation of lymphocytes from human peripheral blood and the intestines as well as the synthesis of interleukin (IL)-2, IL-4, IL-5, and interferon gamma (IFN-γ), but without altering cytokines predominantly expressed by monocytes or macrophages that include IL-1β, IL-6, and IL-8. Screening of a cosmid library from EPEC O127:H6 strain E2348/69 in a laboratory-adapted *E. coli* strain identified a region that conferred inhibitory activity against human peripheral blood mononuclear cells (PBMCs) and the factor responsible was identified by construction of an insertion mutant and named lymphostatin, or lymphocyte inhibitory factor A (LifA) (Klapproth et al., 2000). The *lifA* gene is highly conserved among EPEC and non-O157 EHEC strains.

The *lifA* gene of EPEC strain E2348/69 spans 9,669 bp and is predicted to encode a protein of 365,950 Da (Klapproth et al., 2000). The protein exhibits significant N-terminal homology with large clostridial toxins (LCTs), including a glycosyltransferase motif required for catalytic activity by LCTs (Klapproth et al., 2000). The LCTs from *Clostridium difficile* (toxins A and B; termed TcdA and TcdB, respectively) glucosylate Rho-family GTPases that regulate the actin cytoskeleton, thereby inducing morphological changes and cytotoxicity (Just et al., 1995). LCTs from *C. novyi* (TcnA) and *C. perfringens* (TpeL) transfer N-acetylglucosamine (GlcNAc) to Ras and Rho family GTPases from a uridine diphosphate (UDP) sugar donor (Selzer et al., 1996; Nagahama et al., 2011). Cloning, expression and purification of lymphostatin from EPEC E2348/69 confirmed that it is a 365 kDa protein and established that it inhibits the mitogen-activated proliferation of bovine lymphocytes in the femtomolar range (Cassady-Cain et al., 2016). Substitution of a DTD motif at positions 557-559 of the glycosyltransferase domain for three alanine residues abolished this activity with negligible impact on biophysical properties of the protein (Cassady-Cain et al., 2016). Moreover, lymphostatin was found to bind UDP-GlcNAc in a DTD-dependent manner (Cassady-Cain et al., 2016). However, it remains unclear if this is the only sugar donor bound by lymphostatin, which cellular target(s) is glycosylated, and how this may arrest lymphocyte proliferation.

Lymphostatin also shares a putative cysteine protease motif with LCTs. This motif is characterised by the presence of a catalytic triad that is widely conserved in the C58 family of bacterial virulence factors that contain a YopT-like domain (C1480, H1581, D1596 in EPEC strain E2348/69 LifA) (Bease et al., 2021). A C1480A substitution abolished the ability of lymphostatin to inhibit mitogen-activated proliferation of bovine T cells, without affecting its biophysical properties (Bease et al., 2021). Moreover, lymphostatin was found to be cleaved in treated bovine T lymphocytes and the appearance of an N-terminal c. 140 kDa species was both dependent on C1480A and sensitive to the inhibitors of endosome acidification bafilomycin A1 and chloroquine (Bease et al., 2021). By analogy with LCTs, it has been proposed that lymphostatin enters cells by receptor-mediated endocytosis then undergoes a low pH-dependent conformational change leading to insertion of the protein across the endosome membrane, followed by autocatalytic cleavage to release the catalytic N-terminal domain mediated by the cysteine protease motif (Bease et al., 2021).

Studies using bovine cells have indicated that EPEC lymphostatin is a potent inhibitor of the mitogen- and antigen-stimulated proliferation of T lymphocytes, and to a lesser extent IL-4-stimulated proliferation of B cells, but not natural killer (NK) cells (Cassady-Cain et al., 2017). It was found to affect all T cell subsets (CD4, CD8, WC-1, and γδ T cell receptor) and synthesis of the cytokines IL-2, IL-4, IL-10, IL-17A, and IFN-γ (Cassady-Cain et al., 2017). Even transient exposure of bovine T cells to lymphostatin followed by washing rendered them refractory to mitogenic activation for a least 18 h (Cassady-Cain et al., 2017).

Null *lifA* mutants of EHEC serogroups O5, O26 and O111 are attenuated in their ability to colonise the intestines of calves (Stevens et al., 2002; Deacon et al., 2010). Similarly, lymphostatin was found to be required for colonic colonisation and crypt cell hyperplasia by the murine attaching & effacing pathogen *Citrobacter rodentium* in mice (Klapproth et al., 2005). While the latter study reported that the glycosyltransferase and cysteine protease motifs were required for these phenotypes, subsequent studies established that the deletions caused truncation of the protein and the domains were found to be dispensable during colonisation of calves by EHEC O26:H- (Deacon et al., 2010). The extent to which lymphostatin acts as a colonisation factor by inhibiting lymphocyte function remains unclear. It has been separately reported to influence adhesion (Nicholls et al., 2000), possibly *via* indirect effects on activity of the locus of enterocyte effacement-encoded Type III secretion system (T3SS) (Stevens et al., 2002; Deacon et al., 2010). Moreover, it can be injected into host cells *via* the T3SS, influencing the formation of attaching & effacing lesions by EPEC E2348/69 (Cepeda-Molero et al., 2017). Such injection is not required for inhibition of lymphocyte function however, as the phenotype can be observed with purified protein (Cassady-Cain et al., 2016).

Enteric bacterial pathogens have evolved diverse strategies to interfere with adaptive immunity, including by influencing T lymphocyte metabolism, chemotaxis and apoptosis (reviewed in Cassady-Cain et al., 2018). In relation to the activity of lymphostatin against human cells, studies thus far have primarily used mixed populations of PBMCs (Klapproth et al., 2000) or intraepithelial lymphocytes from surgically resected colon (Klapproth et al., 1996). Moreover, these relied on crude lysates of EPEC rather than highly purified protein. Here we sought to further understand how lymphostatin acts to impair the function of peripheral human T lymphocytes.

## Materials and methods

### Expression and purification of recombinant lymphostatin proteins

Plasmids for the expression of EPEC serotype O127:H6 strain E2348/69 lymphostatin have been described previously (Cassady-Cain et al., 2016). These permit rhamnose-inducible expression of the wild-type protein tagged at the C-terminus with 6xHis for affinity purification (pRham-LifA-6xHis; Cassady-Cain et al., 2016) or the expression of a mutated variant with a substitution in the predicted catalytic motif of the glycosyltransferase domain (pRham-LifA-6xHis DTD-AAA; Cassady-Cain et al., 2016). The pRham vector on which these constructs are based was used as a negative control for plasmid transformation and protein expression.

Each plasmid was transformed into the *E. coli* strain BL21 (DE3) by electroporation. Procedures for protein expression and purification were as described previously with a slight modification (Cassady-Cain et al., 2016). After inducing protein expression with rhamnose, bacteria collected by centrifugation were resuspended in lysis buffer (comprising of 20 mM NaH_2_PO_4_, 300 mM NaCl, 20 mM imidazole, 5% [v/v] glycerol, 0.1% [v/v] Tween-20, 1 mM dithiothreitol, 500 mM non-detergent sulfobetaine 201, ethylenediamine tetraacetic acid (EDTA)-free protease inhibitor cocktail (Roche Molecular Systems, Inc.), 100 µM phenylmethylsulfonyl fluoride (PMSF), and 1 mg/ml lysozyme). Cells were then disrupted by ultrasonication (12 cycles of 10 sec ON/OFF with 60% amplitude; Sonics & Materials, Inc.). After centrifugation at 16,000 x *g* for 20 min, 4°C, the 0.2 µm filtered supernatant containing soluble proteins was processed by immobilized metal affinity chromatography (IMAC) using nickel-charged nitrilotriacetic acid resin (Ni-NTA; Thermo Fisher Scientific) followed by anion exchange chromatography using Q sepharose resin (GE Healthcare). Wild-type lymphostatin (WT-rLifA) and lymphostatin harbouring the DTD substitution (rLifA^DTD-AAA^) were subjected to SDS-PAGE and Western blot analysis. Protein species on the blotted membrane were probed with an anti-His Tag monoclonal antibody (mAb; BioLegend). The same batch of purified recombinant lymphostatin proteins (WT-rLifA and rLifA^DTD-AAA^) was used for all analyses.

### Cell lines and antibodies

The Jurkat T cell line was maintained in the complete Roswell Park Memorial Institute (cRPMI) medium (RPMI-1640 medium supplemented with 10% [v/v] foetal bovine serum (FBS), 20 mM hydroxyethyl piperazineethanesulfonic acid (HEPES), 1 mM sodium pyruvate, 2 mM L-glutamine, and 100 µg/ml penicillin/streptomycin; all reagents were purchased from Gibco) and subcultured every 2-3 days. Cells were washed with cPRMI medium to remove any debris before use in apoptosis assays.

The antibodies used for detection of surface markers, intracellular cytokines, and cell cycle proteins are described in the **Table 1**.

**Table 1:** Antibodies used in this study.

Antibody name	Clone	Manufacturer	Purpose
Purified Mouse Anti-Human CD3	HIT3a	BD Biosciences	Flow cytometric analysis
Purified Mouse Anti-Human CD28	L293	BD Biosciences	
APC Mouse Anti-Human CD3	UCHT1	BD Biosciences	
PerCP Mouse Anti-Human CD4	L200	BD Biosciences	
PE-Cy™7 Mouse Anti-Human CD4	SK3	BD Biosciences	
FITC Mouse Anti-Human CD4	M-T477	BD Biosciences	
APC-Cy™7 Mouse Anti-Human CD8	SK1	BD Biosciences	
FITC Mouse Anti-Human CD8	SK1	BD Biosciences	
FITC Mouse Anti-Human CD69	FN50	BD Biosciences	
PE Rat Anti-Human IL-2	MQ1-17H12	BD Biosciences	
PE Mouse Anti-Human IL-4	3010.211	BD Biosciences	
PE/Cy7 Mouse Anti-Human IFN-γ	B27	BioLegend	
PE Rat Anti-Human IL-10	JES3-9D7	BD Biosciences	
Purified Mouse Anti-Human Retinoblastoma (Rb) protein	G3-245	BD Biosciences	Western blot analysis of cell cycle proteins
Purified Mouse Anti-Cyclin D3	1/Cyclin D3	BD Biosciences	
Purified Mouse Anti-Human Cyclin E	HE12	BD Biosciences	
Purified Mouse Anti-Human Cyclin A	BF683	BD Biosciences	
Purified Mouse Anti-p27^Kip1^	G173-524	BD Biosciences	
Purified Mouse Anti-β-actin	AC-15	GeneTex	
Goat Anti-Mouse IgG (H+L) Polyclonal Antibody, HRP	N/A	Thermo Fisher Scientific	
Ultra-LEAF™ Purified anti-His Tag mAb	J099B12	BioLegend	Western blot analysis of purified LifA proteins

### Isolation of human PBMCs and T lymphocytes

Blood samples were obtained from healthy human donors under approval no. 724/2562(IRB2) from the Siriraj Institutional Review Board (SIRB) committee, Faculty of Medicine Siriraj Hospital, Mahidol University.

Human PBMCs were isolated by density gradient centrifugation. Briefly, heparinized venous blood was diluted with Dulbecco’s phosphate-buffered saline (DPBS) (Lonza) in a ratio of 3:5. Then, 8 ml of the diluted whole blood were gently overlaid onto 3 ml of histopaque^®^1077 (density 1.077 g/ml; Sigma-Aldrich) and centrifuged at 400 x *g* for 30 min at 20°C. PBMCs were collected from the interface and washed twice with DPBS. To lyse red blood cells, the cell pellet was resuspended in 1 ml of 1X lysing buffer (BD Biosciences) and incubated for 5 - 10 min. To stop cell lysis, 2% (v/v) FBS in RPMI medium was then added. Cells were washed and resuspended in cRPMI medium. For flow cytometry analysis of proliferation, cytotoxicity, apoptosis, and cell cycle distribution, the isolated PBMCs were cultured with mitogen and/or tested protein. Subsequently, the cultured PBMCs were stained for cell surface markers of CD4 and CD8 to determine the percentage of T cell subsets.

Human T cells were isolated from PBMCs by negative selection of non-T cells using a pan T-cell isolation kit (Miltenyi Biotec), according to the manufacturer’s instructions. The isolated human T cells were used for Western blot analysis of cell cycle proteins and immunoblot of signaling phosphokinases.

### Stimulation of lymphocytes

Mitogens involved in two distinct pathways of T-cell activation were used in this study; i) an anti-CD3 mAb was used to activate T cells *via* the αβ-T cell receptor (TCR complex) and was combined with an anti-CD28 mAb to provide a co-stimulatory signal (Esensten et al., 2016). Ligation of CD3/CD28 engages TCR signaling (signals one and two); leading to polyclonal proliferation of T cells (Brownlie and Zamoyska, 2013); ii) phorbol 12-myristate 13-acetate (PMA) was used to activate protein kinase C, and was combined with ionomycin, as a calcium ionophore. Together PMA and ionomycin activate T cell proliferation in a way that bypasses the TCR complex (Takahama and Nakauchi, 1996). Concanavalin A (ConA) was used to activate cell expansion in the intracellular cytokine assay and acts by cross-linking components of the TCR complex.

For anti-CD3/anti-CD28 stimulation, unstimulated, untreated, or protein-treated PBMCs or T cells were cultured in a plate with bound anti-CD3 mAb (1 µg/ml; BD Biosciences) containing soluble anti-CD28 mAb (final concentration of 100 ng/ml; BD Biosciences). Plates with bound anti-CD3 mAb were prepared by adding 100 µl of a 1 µg/ml solution of the mAb to the 96-well or 1 ml of a 100 ng/ml solution of the mAb to the 6-well plates. These were stored at 4°C overnight. Before use, the anti-CD3 mAb coated wells were washed twice with DPBS to remove the excess anti-CD3 mAb. ConA (Sigma-Aldrich) was used at a final concentration of 10 µg/ml. PMA and ionomycin (both from Sigma-Aldrich) were used at final concentrations of 20 ng/ml and 500 ng/ml, respectively.

In all experiments, the tested proteins (WT-rLifA and rLifA^DTD-AAA^) including carrier buffer were added only once to cells and were not removed from the cultures until incubation was completed. Carrier buffer was applied for suspending the purified proteins. For cell treatment, the isolated PBMCs and purified T cells were treated with tested proteins or carrier buffer in a separate vial within 1 min and subsequently stimulated with mitogens in a 96-well or 6-well plates at 37°C in a humidified 5% CO_2_ incubator at an indicated time. Unstimulated cells (cells alone; without mitogen and tested protein), untreated cells (cells with mitogen; without tested protein), and carrier buffer-treated cells (cells with mitogen and carrier buffer) were included as controls.

### Analysis of lymphocyte proliferation and cytotoxicity

Isolated PBMCs from blood of healthy donors were labelled with carboxyfluorescein succinimidyl ester (CFSE; Invitrogen) by adding 1 µl of 5 mM of CFSE to 8-10x10^6^ cells/ml and immediately incubated in a 37°C incubator for 10 min. To stop the reaction, cRPMI medium was added to cell suspension, which was then centrifuged at 750 x *g* for 5 min to collect the cells. 3x10^5^ CFSE-labelled cells were treated with WT-rLifA, rLifA^DTD-AAA^, or buffer control in a separate vial. Subsequently, cells were stimulated with anti-CD3/anti-CD28 mAbs or PMA/ionomycin in a 96-well flat bottom plate containing a final volume of 200 µl cRPMI medium/well. Proliferation of CD4^+^ and CD8^+^ T cells was monitored by CFSE partitioning 3 days post-stimulation.

Viobility™ Fixable Dye (Miltenyi Biotec) was used to discriminate between live and dead cells. Cell proliferation and viability were simultaneously assessed by staining the cells with 100 µl of the Viobility™ Fixable Dye (1:1,000 dilution) and incubated in the dark for 30 min. After washing with staining buffer (2% [v/v] FBS and 20 mM EDTA in DPBS), cell surface markers were stained with a mAb cocktail of allophycocyanin (APC)-conjugated CD3 (1:12.5 dilution), phycoerythrin (PE)- Cyanine7 (Cy7)-conjugated CD4 (1:25 dilution), and APC-Cy7-conjugated CD8 (1:25 dilution) and incubated on ice for 30 min in the dark. After washing, cells were resuspended in 200 µl of Cytofix™ fixation buffer (1% [w/v] paraformaldehyde in DPBS; BD Biosciences). The CFSE contents and percentage cell death of CD4^+^ and CD8^+^ T cells were analysed by gating on stained populations using flow cytometry with analysis of data using FlowJo™ Software 10.7.1 (FlowJo, LLC., Oregon, USA).

Proliferation index was determined by counting the total number of divisions divided by the number of cells that have undergone any division. A relative proliferation index was calculated to express the impact of treatments (the ratio of proliferation of the cells treated with mitogen and the tested protein/proliferation of cells treated with mitogen alone). The 50% effective dose (ED_50_) was calculated by using GraphPad Prism software and is the concentration of protein at which mitogen-activated proliferation of the T cells was inhibited by half. Gating of stimulated cells was done on CFSE-proliferating cells using fluorescence compensation of CFSE, whereas fluorescence compensation of live/dead stained populations was gated based on a mixture of stimulated cells (to represent live cells) and cells heated at 56°C for 30 min (to represent dead cells).

### Intracellular cytokine staining

PBMCs (5x10^5^) isolated from healthy donors separately treated with WT-rLifA, rLifA^DTD-AAA^, or carrier buffer were stimulated with anti-CD3/anti-CD28 mAbs, ConA or PMA/ionomycin in a 96-well round bottom plate containing a final volume of 200 µl cRPMI medium/well. For TCR stimulation, ConA was used to stimulate the production of IL-2, IFN-γ, IL-4, while anti-CD3/anti-CD28 mAbs were used to stimulate IL-10 production. For activation that bypasses the TCR complex, PMA/ionomycin was used to stimulate the production of all cytokines. To block the intracellular transport processes within the cells, Brefeldin A (BFA; final concentration 10 µg/ml; Sigma-Aldrich) was added to the wells for IL-2, IFN-γ, and IL-4 detection at 6 h stimulation and further incubated for 12 h. For IL-10 detection, monensin (BD Biosciences) was added after 18 h stimulation and incubated for another 4 h. Dilution of monensin was performed according to manufacturer’s instructions.

CD69 is an early stimulation indicator that can be found on activated lymphocytes (T-, B-, and NK-cells) and neutrophils (Cibrian and Sanchez-Madrid, 2017). CD69 expression appears within one hour after TCR engagement and persists on the cell surface for at least three days (Cassady-Cain et al., 2017). Its functional role involves the regulation of immune responses including T cell proliferation and cytokine secretion (Cibrian and Sanchez-Madrid, 2017). In this study, CD69 expression was assessed in parallel with intracellular cytokine staining. BFA blocks CD69 surface expression mostly on the CD3^+^ human T cells but not its intracellular expression, whereas monensin does not inhibit CD69 expression on the surface of the cells (O’Neil-Andersen and Lawrence, 2002; Lamoreaux et al., 2006). Thus, CD69 was stained intracellularly if BFA was present but was detected on the cell surface marker if monensin was present.

After stimulation, cells were washed with staining buffer then stained for cell surface markers with a cocktail of APC-conjugated anti-CD3 (1:12.5 dilution), peridinin chlorophyll protein complex (PerCP)-conjugated anti-CD4 (1:25 dilution), APC-Cy7-conjugated anti-CD8 (1:25 dilution), fluorescein isothiocyanate (FITC)-conjugated anti-CD69 (1:25 dilution) in the presence of monensin. If BFA was present, cells were stained with the same mAb cocktail for detection of surface markers but without addition of the anti-CD69 mAb. After 30 min incubation on ice, cells were washed twice with staining buffer then fixed and permeabilized with 200 µl of CytoFix/CytoPerm buffer (BD Biosciences) for 20 min on ice. After washing with 1X permeabilization/wash buffer (BD Biosciences), cells were stained for intracellular cytokines as described below.

For IL-2 and IFN-γ, cells were stained with a cocktail of PE-conjugated IL-2, PE-Cy7-conjugated IFN-γ, and FITC-conjugated CD69. For IL-4, a cocktail of PE-conjugated IL-4 and FITC-conjugated CD69 was used. For IL-10, cells were stained with PE-conjugated IL-10 only. A final dilution of 1:25 was used for all antibodies. After incubation, cells were washed and resuspended in 200 µl of Cytofix™ fixation buffer then analysed by flow cytometry. The percentage of cytokine expressing CD69^+^ cells in the CD4^+^ and CD8^+^ T cell populations were analysed by FlowJo™ Software.

Unstimulated PBMCs were used as negative control. For the stimulation control, stimulated cells were cultured without BFA and stained for cell surface markers with a cocktail of mAbs against CD3, CD4, CD8, and CD69. The permeabilization and intracellular staining steps were omitted. For the CD69 intracellular staining control, stimulated cells were cultured in the presence of BFA then stained with a cocktail of antibodies to detect cell surface CD3, CD4, and CD8. After permeabilization, intracellular CD69 was stained. The CD3^+^/CD4^+^ and CD3^+^/CD8^+^ gated events were analysed to assess the percentage of stained CD69 cells.

### Annexin-V apoptosis assay

Annexin V and 7-amino-actinomycin D (7-AAD) were used to distinguish early apoptotic cells from late apoptotic and necrotic cells. Annexin-V is a cellular protein that binds to phosphatidylserine, a marker of the early stages of apoptosis when it is present in the outer leaflet of the plasma membrane (Hasper et al., 2000). The 7-AAD is able to enter cells and intercalate with DNA only upon the loss of membrane integrity in late apoptosis or necrosis (Hasper et al., 2000).

In brief, 3x10^5^ PBMCs isolated from healthy donors were mixed with WT-rLifA, rLifA^DTD-AAA^, or carrier buffer followed by anti-CD3/anti-CD28 stimulation for 48 h in a 96-well flat bottom plate containing a final volume of 200 µl cRPMI medium/well. After washing, the stimulated cells were stained for cell surface markers with a cocktail of APC-conjugated anti-CD3 (1:12.5 dilution), PE-Cy7-conjugated anti-CD4 (1:25 dilution), and FITC-conjugated anti-CD8 (1:25 dilution) and incubated on ice for 30 min. Cells were washed with staining buffer followed by ice-cold annexin V binding buffer and resuspended with 100 µl of binding buffer, then added 5 µl of each PE-conjugated annexin-V and 7-AAD solution to the suspension (BD Biosciences). Tubes were placed on ice for 15 min in the dark then 400 µl of binding buffer was added to the suspension. Cells were analysed by flow cytometry. Early, late apoptotic and necrotic cells were quantified following quadrant statistics analysis using FlowJo™ software. The annexin-V^+^/7-AAD^+^ stained cells were considered to be late apoptotic and necrotic phases. The annexin-V^+^/7-AAD^-^ stained cells was considered as early apoptotic cells and the annexin-V^-^/7-AAD^-^ stained cells was considered to be non-apoptotic.

As positive controls for apoptosis and necrosis, Jurkat cells were cultured for 5-6 h with 5 µM camptothecin, a topoisomerase inhibitor, and the stimulated PBMCs were treated with 11.5 µM camptothecin for 18 h. Histogram markers were set based on the unstimulated cells in conjunction with apoptosis and necrosis controls.

### Cell cycle analysis

Analysis of the proportion of cells in different phases of the cell cycle was carried out by using propidium iodide (PI) staining of DNA content. To allow cells to go into G0/G1 phase, the isolated PBMCs from healthy donors were starved overnight in serum-free RPMI medium supplemented with 20 mM HEPES buffer. The PBMCs were washed out serum-free medium then suspended in cRPMI medium containing FBS to allow cells to be released into cell cycle. Subsequently, 2x10^6^ of the PBMCs were treated with WT rLifA, rLifA^DTD-AAA^, or carrier buffer followed by stimulation with anti-CD3/anti-CD28 mAbs for 48 h in a 6-well plate containing a final volume of 2 ml cRPMI medium/well. For detection of CD4^+^ and CD8^+^ T cells, the cells were seeded in a separate well and staining of cell surface CD4 and CD8 was done in a separate tube.

After stimulation, PBMCs were washed and stained with FITC-conjugated anti-CD4 or FITC-conjugated anti-CD8 at 1:10 dilutions of each. After placing on ice for 30 min, cells were washed and resuspended in 300 μl of ice-cold DPBS then 700 μl of ice-cold absolute ethanol were slowly added in a dropwise manner to the cell suspension with continual mixing. The fixed cells were stored at -20°C for 2 h then washed and treated with 200 μl of staining buffer containing PI (final concentration of 40 µg/ml; Sigma-Aldrich) and ribonuclease A (final concentration of 100 - 200 μg/ml; Sigma-Aldrich) for 45 min in the dark. The DNA content of CD4^+^ and CD8^+^ T cells was analysed by flow cytometry. Cell cycle distribution was analysed by using FlowJo™ Software.

### Western blot analysis of cyclins and cell cycle regulatory proteins

Isolated PBMCs were rested overnight in the serum-free RPMI medium. On the following day, T cells were isolated to a purity of 95-99% by using the Pan T cell isolation kit. The purified T cells were stained for 10 min with PI (final concentration of 20 µg/ml) to assess viability. Flow cytometry was used to determine purity and viability of purified T cells. In a similar way to the cell cycle analysis, the serum-deprived T cells were washed and released into the cell cycle by resuspending in cRPMI medium containing FBS. Purified T cells (2 - 2.5x10^6^) T cells were treated with 10 ng/ml of WT-rLifA or left untreated then stimulated with anti-CD3/anti-CD28 mAbs for 24, 48 or 72 h in a 6-well plate containing a final volume of 2 ml cRPMI medium/well.

T cells were lysed in 60 µl of NP-40 lysis buffer (comprising of 50 mM Tris-HCl pH 7.4, 250 mM NaCl, 5 mM EDTA, 1% [v/v] IGEPAL CA-630 (Sigma Aldrich), 1 mM PMSF, EDTA-free protease inhibitor cocktail and phosphatase inhibitor cocktail (Thermo Scientific) for 30 min on ice with intermittent vortexing. Following centrifugation at 16,000 x *g* for 10 min at 4°C, the supernatants were collected and stored at -70°C. Total protein of cell lysates was measured using the Pierce™ BCA Protein Assay Kit (Thermo Fisher Scientific).

For Western blot analysis, cell lysates were denatured with Laemmli SDS sample buffer (BioRad) at 95°C for 5 min then analysed on a 4-15% Tris-Glycine gradient gel (BioRad), and electro-transferred to 0.2 µm nitrocellulose membranes (Invitrogen). Total protein was loaded at 10 µg for detection of phosphorylated retinoblastoma (pRb) and β-actin, 20 µg for cyclin D3, cyclin E, and p27^Kip1^ detection. Detection of β-actin was used as loading control. The blotted membrane was blocked with 5% (w/v) bovine serum albumin (BSA) in Tris-Buffered Saline (TBS) pH 7.4 containing 0.02% (v/v) Tween-20 (TBS-T) for 1 h, then incubated overnight at 4°C with diluted primary antibody in 3% (w/v) BSA/TBS-T; anti-Rb mAb (1:1,000), anti-cyclin D3 mAb (1:1,000), anti-cyclin E mAb (1:500), anti-cyclin A mAb (1:500), anti- p27^Kip1^ mAb (1:400), or anti-β-actin mAb (1:5,000). The membranes with bound antibodies were incubated with diluted goat anti-mouse IgG (H+L), horseradish peroxidase (HRP) conjugate at 1:2,000 dilution for 2 h. Blots were washed three times with TBS-T between each step, then visualized by SuperSignal™ West Pico PLUS Chemiluminescent Substrate (Thermo Fisher Scientific). Images were captured using a ChemiDoc™ Gel Imaging System (BioRad).

For densitometric analysis, signal intensity was quantified using Image Lab software (BioRad) and normalized to β-actin. The relative signal intensity was expressed as fold-change relative to the β-actin control.

### Immunoblot of signaling phosphokinases

Isolated human PBMCs were serum-deprived overnight and subsequently T cells were isolated to a purity of 95-99% by using the Pan T cell isolation kit. Purity and viability of the purified T cells were assessed by flow cytometry as for Western blot analysis of cyclins. Approximately 6x10^6^ serum-starved T cells were treated with 10 ng/ml of WT-rLifA or left untreated for 1 h followed by anti-CD3/anti-CD28 stimulation for 15 min in a 6-well plate containing a final volume of 2 ml cRPMI medium/well. Levels of human protein kinase phosphorylation was determined using the Proteome Profiler Human Phospho-Kinase Array Kit (R&D Systems). All procedures were performed according to the manufacturer’s instructions. Stimulated T cells were lysed and 150 µg of total protein lysates were applied to nitrocellulose membranes containing capture antibodies specific to protein kinases as a series of separate spots. Images were captured by ChemiDoc™ Gel Imaging System (BioRad). Pixel density in each spot of the array was quantified by using Image Lab software (BioRad). The relative signal intensity was expressed as fold-change relative to the untreated cells.

### Statistical analysis

Data are expressed as the mean ± standard error of the mean (S.E.M.) of replicated measurements, as indicated in Figure legends. All statistics were carried-out using GraphPad Prism 7.05 (GraphPad Software Inc., La Jolla, California, USA). The Kruskal-Wallis one-way ANOVA with Dunn’s multiple comparison was used to compare responses to different mitogens or different concentrations of tested proteins. Spearman’s rank correlation test was performed to examine the correlation between proliferation and cell cycle distributions in G0/G1 or S phases. *p* values < 0.05 were considered to be statistically significant.

## Results

### Expression and purification of recombinant lymphostatin proteins

Plasmids for the expression of wild-type lymphostatin from EPEC strain E2348/69 (WT-rLifA) or the DTD substitution mutant of lymphostatin (rLifA^DTD-AAA^) were transformed into *E. coli* BL21 (DE3) and verified by PCR and agarose gel electrophoresis (data not shown). The nucleotide sequence of the DTD-AAA substitution contains a *Not*I restriction site and fragments of the expected size were detected upon *Not*I digestion of the *lifA* amplicon (Cassady-Cain et al., 2016). Following purification by affinity chromatography, the WT-rLifA and rLifA^DTD-AAA^ proteins were analysed by SDS-PAGE and Western blotting and confirmed to have a molecular weight of ~365 kDa (**Figure S1**) as expected (Cassady-Cain et al., 2016).

### Lymphostatin inhibits proliferation of anti-CD3/anti-CD28-stimulated human T lymphocyte subsets in a manner that requires a DTD motif and without affecting cell viability

It is well-established that whole-cell lysates of EPEC E2348/69 or *E. coli* K-12 encoding LifA on a cosmid inhibit activation of bulk human PBMCs by ConA or pokeweed mitogen (Klapproth et al., 2000). In addition, lymphostatin likely affects early signaling molecules, as shown inhibition of T cell proliferation in bovine *via* ConA stimulation, but not PMA/ionomycin (Cassady-Cain et al., 2017). We queried whether highly purified lymphostatin was able to inhibit proliferation of human T lymphocytes stimulated *via* cross-linking of the TCR and CD28, or when using PMA/ionomycin that mimics TCR and co-receptor activation, but in a way that bypasses membrane receptor signaling.

**Figure 1:**
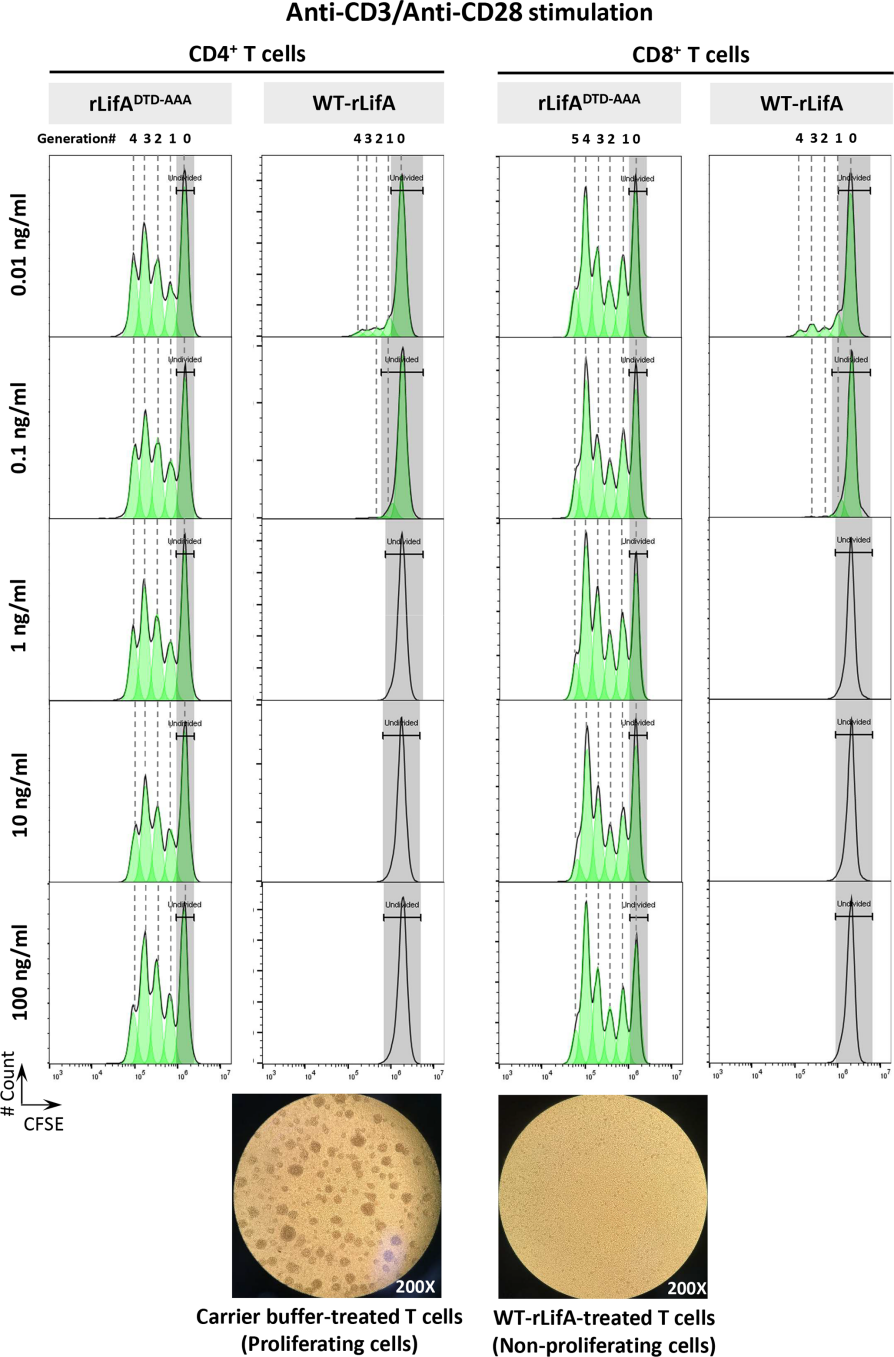
Lymphostatin inhibits proliferation of human CD4^+^ and CD8^+^ T cells in a manner dependent on its DTD motif. Upper panel shows CFSE content of the anti-CD3/anti-CD28-stimulated CD4^+^ and CD8^+^ T cells in the presence of rLifA^DTD-AAA^ and WT-rLifA. The CD4^+^ and CD8^+^ T cell populations were determined by gating on CD3^+^/CD4^+^ and CD3^+^/CD8^+^, respectively. Lower panel shows a representative light micrograph of the anti-CD3/anti-CD28-stimulated T lymphocytes 3 days after treatment with carrier buffer (left) or 10 ng/ml of WT-rLifA (right) (200X magnification). Data are representative of one sample.

**Figure 2:**
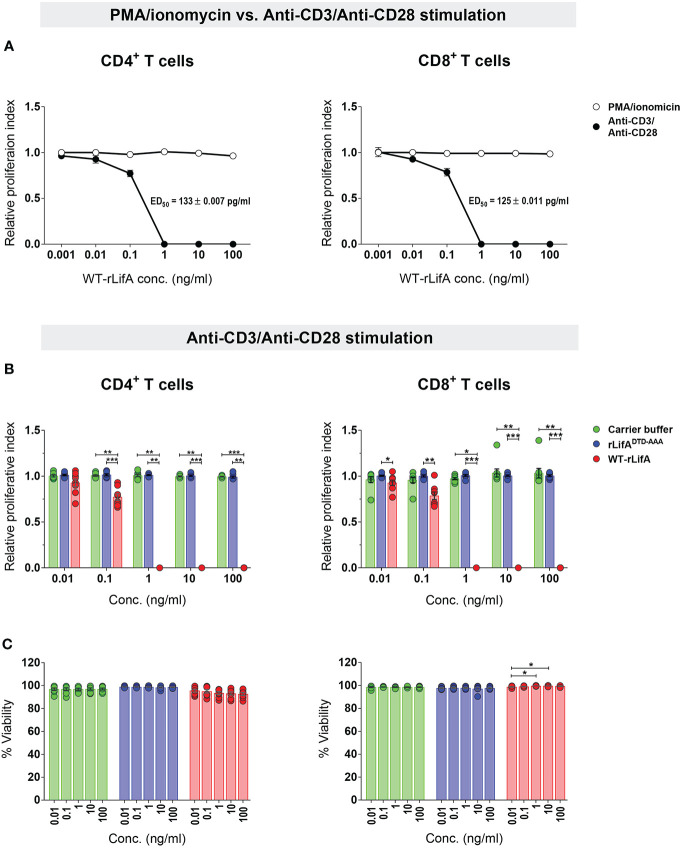
Lymphostatin inhibits proliferation of human CD4^+^ and CD8^+^ T cells without affecting viability. **(A)** Relative proliferation index of CD4^+^ and CD8^+^ T cells treated with varied WT-rLifA concentrations was compared between stimulation with anti-CD3/anti-CD28 mAbs versus PMA/ionomycin. The ED_50_ of WT-rLifA against CD4^+^ and CD8^+^ T cells treated with WT-rLifA is shown. **(B)** Relative proliferation index of anti-CD3/anti- CD28-stimulated CD4^+^ and CD8^+^ T cells in the presence of WT-rLifA, rLifA^DTD-AAA^, or carrier buffer. **(C)** Viability of anti-CD3/anti-CD28-stimulated CD4^+^ and CD8^+^ T cells in the presence of WT-rLifA, rLifA^DTD-AAA^, or carrier buffer. Data represent the mean from 8 healthy donors ± S.E.M. *p < 0.05, **p < 0.01, ***p < 0.001 using Kruskal-Wallis test followed by Dunn’s test. Symbols with circles in **Figures 2B, C** represent data from the individual donors.

Our results demonstrated that WT-rLifA inhibited proliferation of anti-CD3/anti-CD28-stimulated human CD4^+^ and CD8^+^ T cell subsets in a concentration-dependent manner (**Figures 1**, **2A, 2B**), but did not impair proliferation stimulated with PMA/ionomycin (**Figure 2A**). The ED_50_ of WT-rLifA was 133 ± 0.007 pg/ml and 125 ± 0.011 pg/ml for CD4^+^ and CD8^+^ human T cells, respectively. This compares to previously reported ED_50_ values for WT-rLifA of 25 ± 4.6 pg/ml (Cassady-Cain et al., 2016) and 54 ± 19 pg/ml (Cassady-Cain et al., 2017) when using bovine T cells. The data are also consistent with evidence that LifA has a global effect across T cell subsets from cattle at the level of membrane proximal signaling (Cassady-Cain et al., 2017).

Since substitution of a DTD motif within a predicted glycosyltransferase domain results in loss of lymphostatin activity against bovine T cells (Cassady-Cain et al., 2016), we investigated whether the DTD motif is required for inhibition of proliferation of human T cells. The anti-CD3/anti-CD28-stimulated CD4^+^ and CD8^+^ T cells treated with rLifA^DTD-AAA^ showed a proliferation pattern similar to carrier buffer-treated cells, indicating a complete loss of inhibitory activity (**Figures 2B**). There was no statistically significant difference in the proliferation of CD4^+^ and CD8^+^ T cells between rLifA^DTD-AAA^ and buffer treatment (**Figure 2B****)**.

Following anti-CD3/anti-CD28 stimulation, the lowest concentration of WT-rLifA resulting in a significant difference in CD4^+^ T cell proliferation when compared to buffer control or rLifA^DTD-AAA^ treatment was 0.1 ng/ml (**Figure 2B**). For CD8+ T cells, the lowest concentration of WT-rLifA resulting in a significant difference from rLifA^DTD-AAA^ was 0.01 ng/ml (**Figure 2B**). However, WT-rLifA at 1 - 100 ng/ml impeded proliferation of both CD4^+^ and CD8^+^ T cells (**Figures 1**, **2A, 2B**). Conversely, neither treatment with buffer nor with all concentrations tested (0.01 – 100 ng/ml) of rLifA^DTD-AAA^ impaired the proliferation of CD4^+^ and CD8^+^ T cells (**Figures 1**, **2B**).

To determine if the effect of WT-rLifA on proliferation could be explained by direct cytotoxicity, we examined the viability of treated cells. Our results showed that cell viability was in a range of 86.4%-99.55% for CD4^+^ and 97.33%-99.94% for CD8^+^ upon WT-rLifA treatment at concentrations of 0.01 to 100 ng/ml (**Figure 2C**). There was no significant difference in the percentage of viable CD4^+^ T cells across the WT-rLifA concentrations tested, while a slight but significant difference in viable CD8^+^ T cells were observed when comparing the effect of some concentrations of WT-rLifA (0.01 vs. 1 ng/ml; *p* = 0.0285 and 0.01 vs. 10 ng/ml; *p* = 0.0275). Viability of CD4^+^ and CD8^+^ T cell did not show any statistical difference among all tested concentrations of rLifA^DTD-AAA^ and buffer (**Figure 2C**). At concentrations of WT-rLifA at which CD4^+^ and CD8^+^ T lymphocyte proliferation is inhibited, we can conclude that there was no significant loss of cell viability.

### Lymphostatin inhibits cytokine production of anti-CD3/anti-CD28-stimulated human T lymphocyte subsets in a manner dependent on its DTD motif

Previous studies have shown that crude lysates of *E. coli* containing lymphostatin suppressed secretion of IL-2, IL-4, and IFN-γ in bulk human PBMCs (Klapproth et al., 2000). In the present study, we sought to investigate the effect of purified lymphostatin on cytokine production in human T cells by using intracellular cytokine staining to observe expression at a single-cell level. Based on the results of CFSE proliferation, the significant difference in T cell proliferation was seen at 0.1 ng/ml of WT-rLifA and the maximal inhibitory effect was observed at 1 ng/ml. Therefore, we chose these two concentrations to study the impact of lymphostatin on cytokine production by human T cells. The rLifA^DTD-AAA^ mutant was also examined to determine if observed effects are dependent on the DTD glycosyltransferase motif. Expression of CD69, a surrogate marker of T cell responsiveness, was also used to monitor the activated T cells.

**Figure 3:**
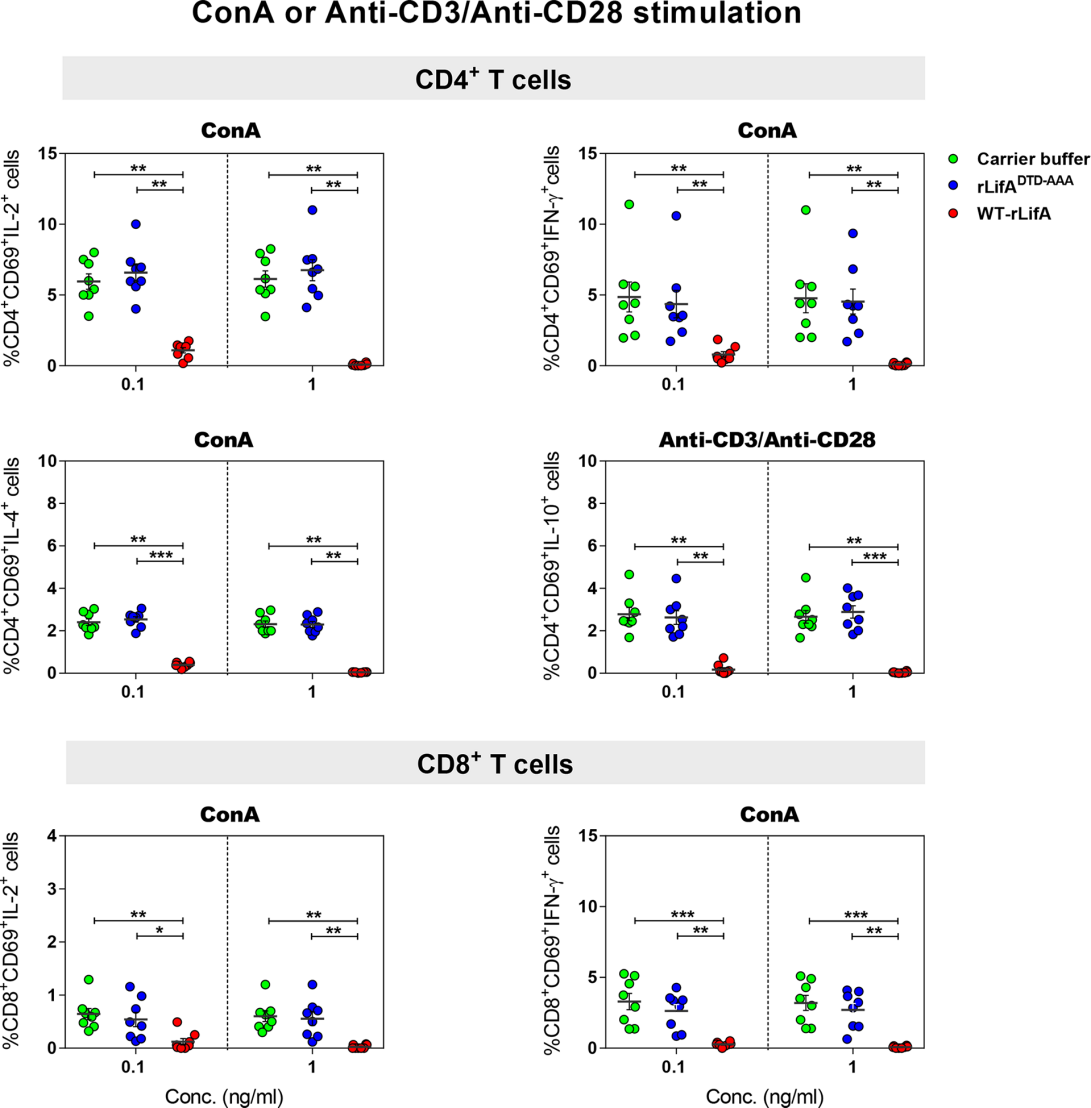
Lymphostatin inhibits intracellular cytokine production in anti-CD3/anti-CD28 or ConA-stimulated CD4^+^ and CD8^+^ T cells. Dot plots show the percentage of CD4^+^/CD69^+^ cells producing IL-2, IFN-γ, IL-4, IL-10 and CD8^+^/CD69^+^ cells producing IL-2, IFN-γ in the presence of WT-rLifA, rLifA^DTD-AAA^, or carrier buffer. Data represent the mean from 8 healthy donors ± S.E.M. *p < 0.05, **p < 0.01, ***p < 0.001 using Kruskal-Wallis test followed by Dunn’s test.

Following anti-CD3/anti-CD28 activation, our results showed that WT-rLifA at 0.1 and 1 ng/ml significantly inhibited cytokine production in CD4^+^ and CD8^+^ T cells. In the presence of WT-rLifA at 0.1 and 1 ng/ml, the percentage of IL-2-, IL-4-, IFN-γ-, and IL-10-producing CD4^+^ T cells were significantly reduced when compared to cells treated with the carrier buffer or rLifA^DTD-AAA^ (**Figure 3**). Similar observations were made for IL-2- and IFN-γ-producing CD8^+^ T cells (**Figure 3**). This is consistent with the capacity of WT-rLifA to inhibit secretion of IL-2, IL-4, IL-10 and IFN-γ by bovine T cells at these concentrations (Cassady-Cain et al., 2017). In contrast, no statistical difference of the percentage of cytokine-producing cells was detected between CD4^+^ or CD8^+^ T cells treated with rLifA^DTD-AAA^ at both 0.1 and 1 ng/ml relative to cells treated with carrier buffer (**Figure 3**).

**Figure 4:**
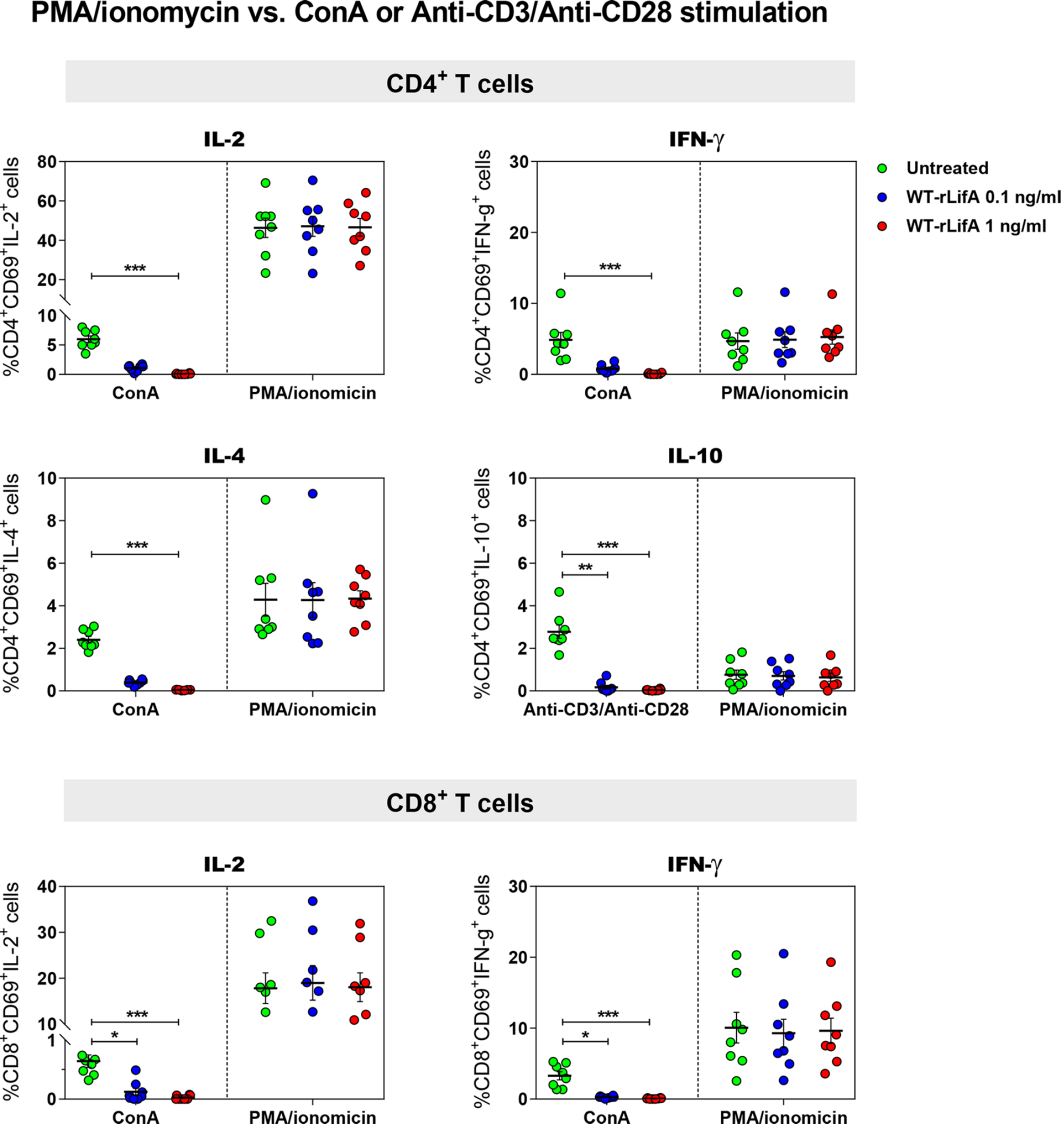
Lymphostatin did not inhibit intracellular cytokine production in PMA/ionomycin-stimulated CD4^+^ and CD8^+^ T cells. Dot plots show the percentage of CD4^+^/CD69^+^ cells producing IL-2, IFN-γ, IL-4, IL-10 and CD8^+^/CD69^+^ cells producing IL-2, IFN-γ in the presence of WT-rLifA or left untreated (cells with mitogen; no tested protein) and compared between anti-CD3/anti-CD28 and PMA/ionomycin stimulation. Data represent the mean from 8 healthy donors ± S.E.M. *p < 0.05, **p < 0.01, ***p < 0.001 using Kruskal-Wallis test followed by Dunn’s test.

Following PMA/ionomycin stimulation, WT-rLifA did not affect cytokine production in CD4^+^ and CD8^+^ T cells, as there were no significant changes in the percentage of cells expressing the cytokines analysed in CD4^+^ and CD8^+^ T cells in the presence of WT-rLifA at 0.1 or 1 ng/ml (**Figure 4**). This is consistent with the lack of inhibition of proliferation of PMA/ionomycin-activated cells (**Figure 2A**).

For cytokine production by T helper (Th) cell subpopulations, it is known that IL-2 and IFN-γ are secreted by type 1 Th (Th1) cells, IL-4 is secreted by type 2 Th (Th2) and in some cases, IL-10 is secreted by regulatory T cells (Treg), whereas IL-2 and IFN-γ are also secreted by CD8^+^ T cells. Our data suggest that lymphostatin can suppress cytokine production by the major human CD4^+^ Th subsets and CD8+ T cells following activation by anti-CD3/anti-CD28 or ConA, but not PMA/ionomycin. Moreover, this is the first report to show that the DTD glycosyltransferase motif is required for suppression of cytokine production in human T cells by lymphostatin.

### Lymphostatin suppresses early T cell activation

**Figure 5:**
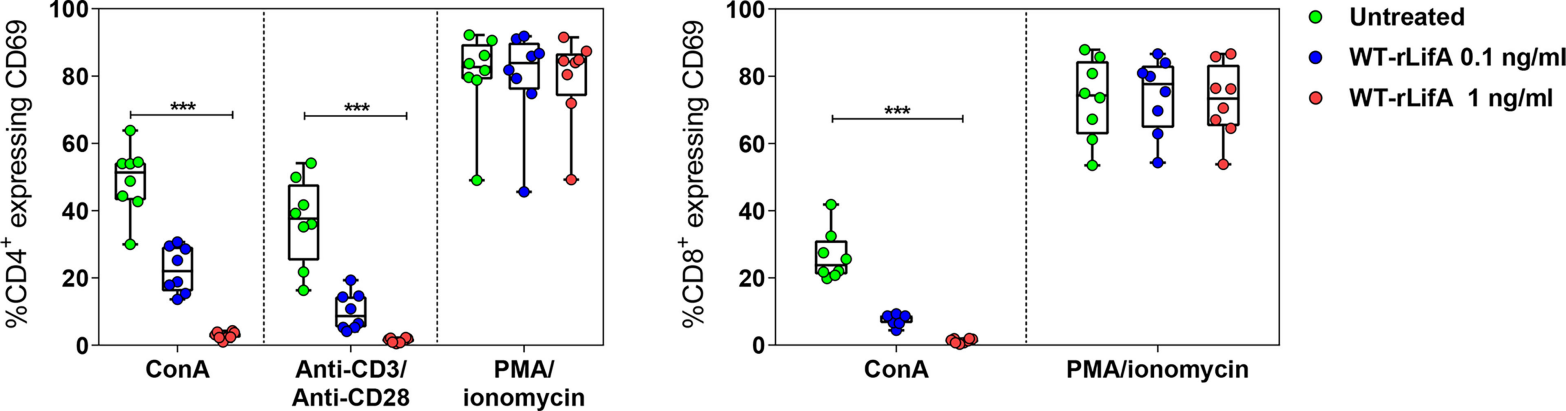
Lymphostatin suppresses CD69 expression in anti-CD3/anti-CD28 or ConA-stimulated CD4^+^ and CD8^+^ T cells. Box and whisker plots show the percentage of CD69 expression on anti-CD3/anti-CD28, ConA, and PMA/ionomycin-stimulated CD4^+^ T cells and ConA and PMA/ionomycin-stimulated CD8^+^ T cells treated with WT-rLifA, or left untreated (cells with mitogen; no tested protein). Data represent the mean from 8 healthy donors. Symbols with circles represent data from the mean of technical replicates for each of the 8 donors. ***p < 0.001 using Kruskal-Wallis test followed by Dunn’s test.

**Figure 6:**
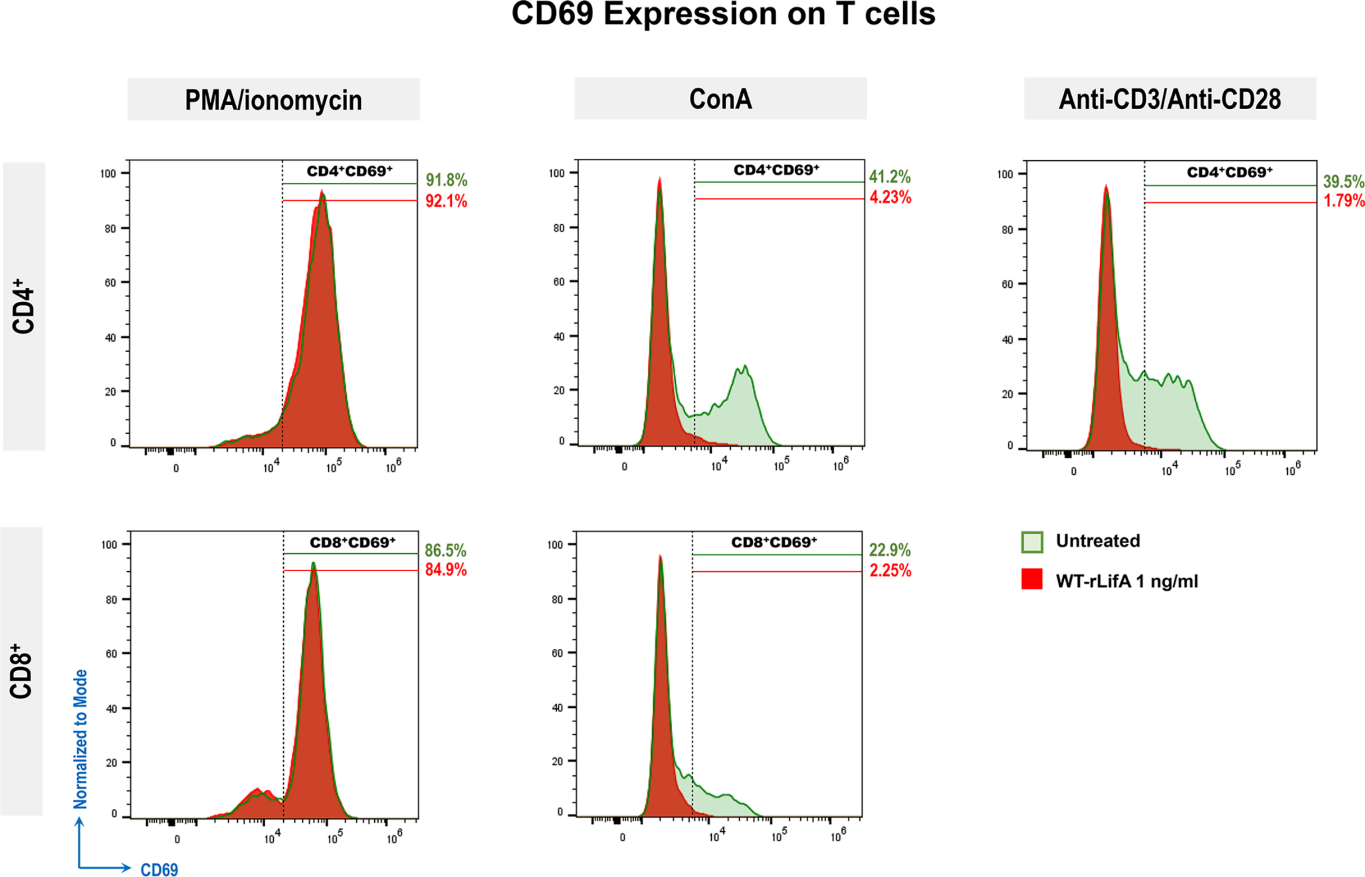
Representative histograms show CD69 expression (normalized to mode) of PMA/ionomycin, ConA, or anti-CD3/anti-CD28-stimulated CD4^+^ and CD8^+^ T cells comparing between untreated cell versus WT-rLifA (1 ng/ml) treatment. For each histogram, % is the percentage of CD69 expression on PMA/ionomycin, ConA, or anti-CD3/anti-CD28-stimulated CD4^+^ or CD8^+^ T cells upon WT-rLifA treatment (shaded areas in red) or left untreated (shaded areas in green). Histograms represent data from a technical replicate of one donor.

A study using the *Helicobacter pylori* exotoxin Vacuolating Toxin A (VacA) reported that it blocked TCR-dependent T cell activation by completely inhibiting CD69 expression on anti-CD3-stimulated human CD4^+^ and CD8^+^ peripheral blood lymphocytes (Boncristiano et al., 2003). Upon anti-CD3/anti-CD28 or ConA stimulation, we observed that percentage of CD4^+^ and CD8^+^ T cells expressing CD69 was significantly reduced following WT-rLifA treatment (1 ng/ml) compared to untreated cells (**Figure 5**). WT-rLifA at 1 ng/ml fully suppressed CD69 expression in CD4^+^ **(** **Figures 5**, **6****)** and CD8^+^ T cells (**Figures 5**, **6**). Additionally, the significant reductions of CD4^+^/CD69^+^ cells producing IL-2, IFN-γ, IL-4, IL-10 and CD8^+^/CD69^+^ cells producing IL-2, IFN-γ in the presence of 1 ng/ml of WT-rLifA were found as compared to PMA/ionomycin stimulation. In other words, the levels of CD69 expression were concordant with the production of intracellular cytokines. Conversely, CD69 expression in the PMA/ionomycin stimulated-CD4^+^ and CD8^+^ T cells did not alter regardless of the WT-rLifA concentration used (**Figures 5**, **6**). Overall, these data indicate that lymphostatin acts at an early stage of lymphocyte activation.

### Lymphostatin did not induce apoptosis and necrosis of anti-CD3/anti-CD28-stimulated human T lymphocyte subsets

To examine whether lymphostatin induced apoptosis or necrosis in CD4^+^ and CD8^+^ human T cells, we used a combination of annexin-V and 7-AAD to stain the T cells activated with anti-CD3/anti-CD28 and treated with WT-rLifA.

**Figure 7:**
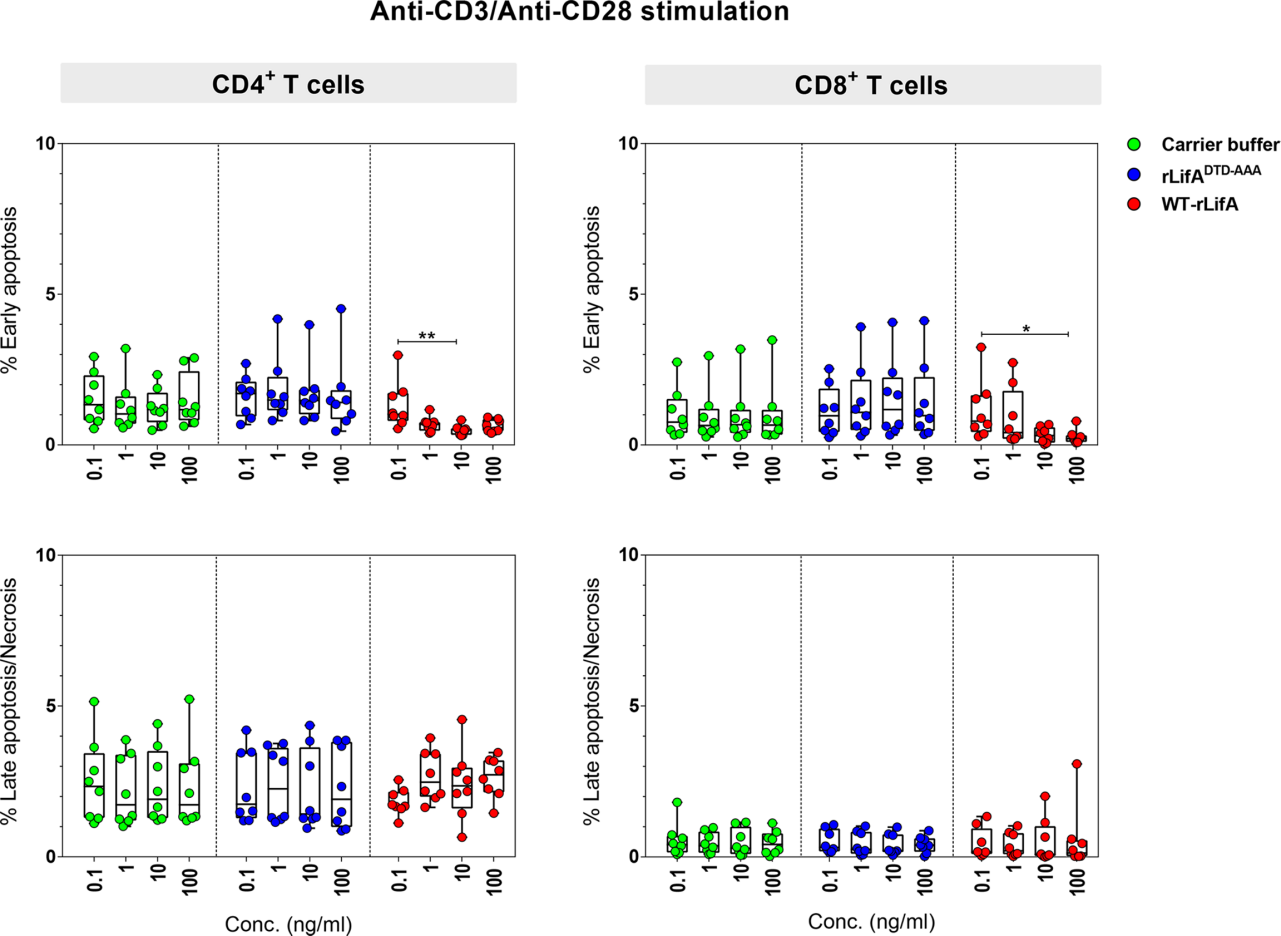
Lymphostatin induces neither apoptosis nor necrosis in anti-CD3/anti-CD28-stimulated CD4^+^ and CD8^+^ T cells. Box and whisker plots show the percentage of early apoptosis, and mixture of late apoptosis and necrosis in anti-CD3/anti-CD28-stimulated CD4^+^ and CD8^+^ T cells treated with WT-rLifA, rLifA^DTD-AAA^, or carrier buffer. Data represent the mean from 8 healthy donors. Symbols with circles represent data from the individual donors. *p < 0.05, **p < 0.01 using Kruskal-Wallis test followed by Dunn’s test.

In the presence of WT-rLifA, the percentages of early apoptotic, late apoptotic, and necrotic cells of anti-CD3/anti-CD28-stimulated CD4^+^ and CD8^+^ T cells were low number in all tested concentrations (0.1-100 ng/ml) (**Figure 7**). The percentage of cells in early apoptosis ranged between 0.31%-2.98% for CD4^+^ and 0.02%-3.24% for CD8^+^ T cells. Similarly, the percentage of cells in late apoptosis and necrosis were in a range of 0.65%-4.55% for CD4^+^ and 0.02%-3.08% for CD8^+^ T cells (**Figure 7**). Following WT-rLifA treatment, the percentage of cells in early apoptosis showed a significant difference only between 0.1 vs. 10 ng/ml WT-rLifA for CD4^+^ T cells and 0.1 vs. 100 ng/ml WT-rLifA for CD8^+^ T cells however, the percentage of early apoptotic T cells of both CD4^+^ and CD8^+^ treated with high WT-rLifA concentrations (10 and 100 ng/ml) was less than that treated with lower WT-rLifA concentration of 0.1 ng/ml (**Figure 7**).

**Figure 8:**
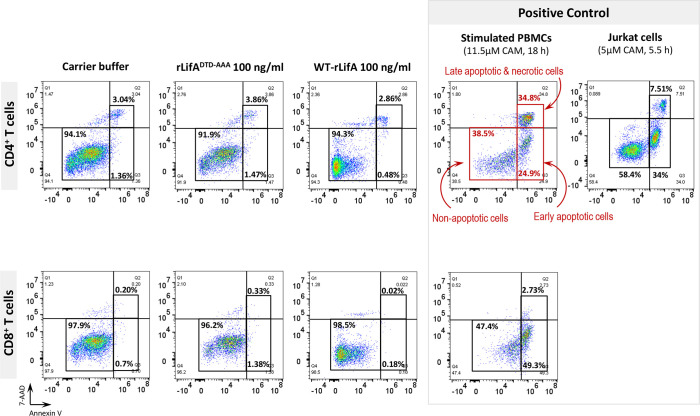
Flow cytometric dot plots represent the percentage of non-apoptotic, early apoptotic, and mixture of late apoptotic and necrotic CD4^+^ and CD8^+^ T cells treated with WT-rLifA, rLifA^DTD-AAA^, or carrier buffer. Data are representative of one sample. The anti-CD3/anti-CD28-stimulated CD4^+^ and CD8^+^ T cells treated with 11.5 µM camptothecin for 18 h and Jurkat T cell line treated with 5 µM camptothecin for 5.5 h were used as positive controls of apoptosis and necrosis. CAM; Camptothecin.

The percentage of necrotic CD4^+^ and CD8^+^ T cells was not significantly increased at all WT-rLifA concentrations tested (**Figure 7**). This observation was similar to T cells treated with carrier buffer or rLifA^DTD-AAA^. Even at a high concentration of 100 ng/ml WT-rLifA, 100-fold of the dose that gives maximal inhibitory effect of proliferation, we observed no increase the extent of apoptosis and necrosis in anti-CD3/anti-CD28-stimulated CD4^+^ and CD8^+^ T cells (**Figures 7**, **8**). For positive controls, the topoisomerase inhibitor camptothecin substantially induced high levels of apoptosis in both Jurkat cells and CD4^+^ human T cells, but a minimal level of late apoptosis/necrosis was detected in the camptothecin-treated CD8^+^ T cells (**Figure 8**).

Overall, these findings suggests that lymphostatin does not induce apoptosis or necrosis in the major human T cell subsets at concentrations sufficient to inhibit their proliferation and cytokine production.

### Lymphostatin induces G0/G1 arrest in anti-CD3/anti-CD28–stimulated human CD4^+^ and CD8^+^ T- lymphocyte subsets in a manner dependent on its DTD motif

We hypothesized that the inhibition of lymphocyte proliferation by lymphostatin may be associated with arrest of the cell cycle at a specific phase. Therefore, we initially examined the DNA content of anti-CD3/anti-CD28-stimulated CD4^+^ and CD8^+^ T cells in the presence of WT-rLifA, rLifA^DTD-AAA^ or carrier buffer.

**Figure 9:**
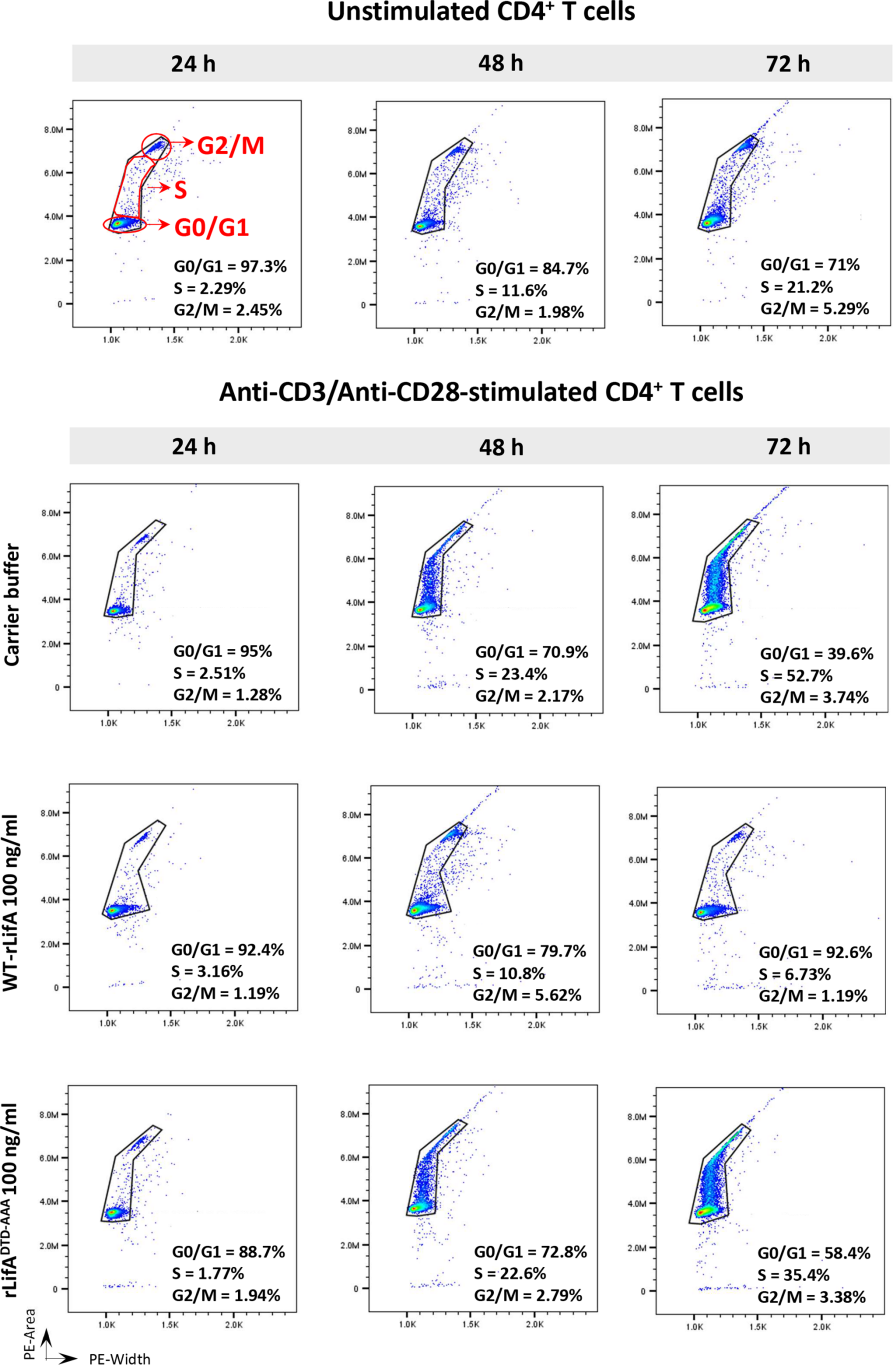
Lymphostatin arrests the cell cycle in G0/G1 phase in anti-CD3/anti-CD28-stimulated CD4^+^ T cells throughout 72 h incubation period. Histograms represent the DNA contents of G0/G1, S, and G2/M phases in the unstimulated (without treatment) and anti-CD3/anti-CD28-stimulated CD4^+^ T cells treated with WT-rLifA, rLifA^DTD-AAA^, or carrier buffer at incubation time of 24, 48, and 72 h. Representative data using cells from one donor are shown.

Cell cycle progression of CD4^+^ T cells was first studied using T cells from a single healthy donor following treatment with 100 ng/ml of WT-rLifA, rLifA^DTD-AAA^, or buffer and subsequent stimulation with anti-CD3/anti-CD28 for 24, 48, and 72 h. CD4^+^ T cells treated with carrier buffer or rLifA^DTD-AAA^ had normal patterns of cell cycle progression as detected by flow cytometric analysis of DNA content (**Figure 9**). The CD4^+^ T cells exhibited notable DNA synthesis in S phase after 48 and 72 h stimulation, with the percentage of cells in S phase increasing from 23.4% (48 h) to 52.7% (72 h) in the buffer treatment, and moderately increased from 22.6% (48 h) to 35.7% (72 h) in the rLifA^DTD-AAA^ treatment. The percentage of cells in the G0/G1 phase was gradually reduced in proportion to increasing stimulation time; from 95% to 39.6% (24 to 72 h) for buffer and from 88.7% to 58.4% (24 to 72 h) for the rLifA^DTD-AAA^ treatment. Conversely, the percentage of CD4^+^ T cells in S phase following treatment with 100 ng/ml WT-rLifA-treated remained low throughout 72 h stimulation (between 3.16% to 6.73% at 24 to 72 h) but the percentage of cells in G0/G1 phase was between 92.4% to 92.6% (24 to 72 h). A similar observation was also seen in the unstimulated cells but the percentage of cells in S and G0/G1 phases were slightly higher than WT-rLifA treatment at 72 h stimulation (**Figure 9**).

**Figure 10:**
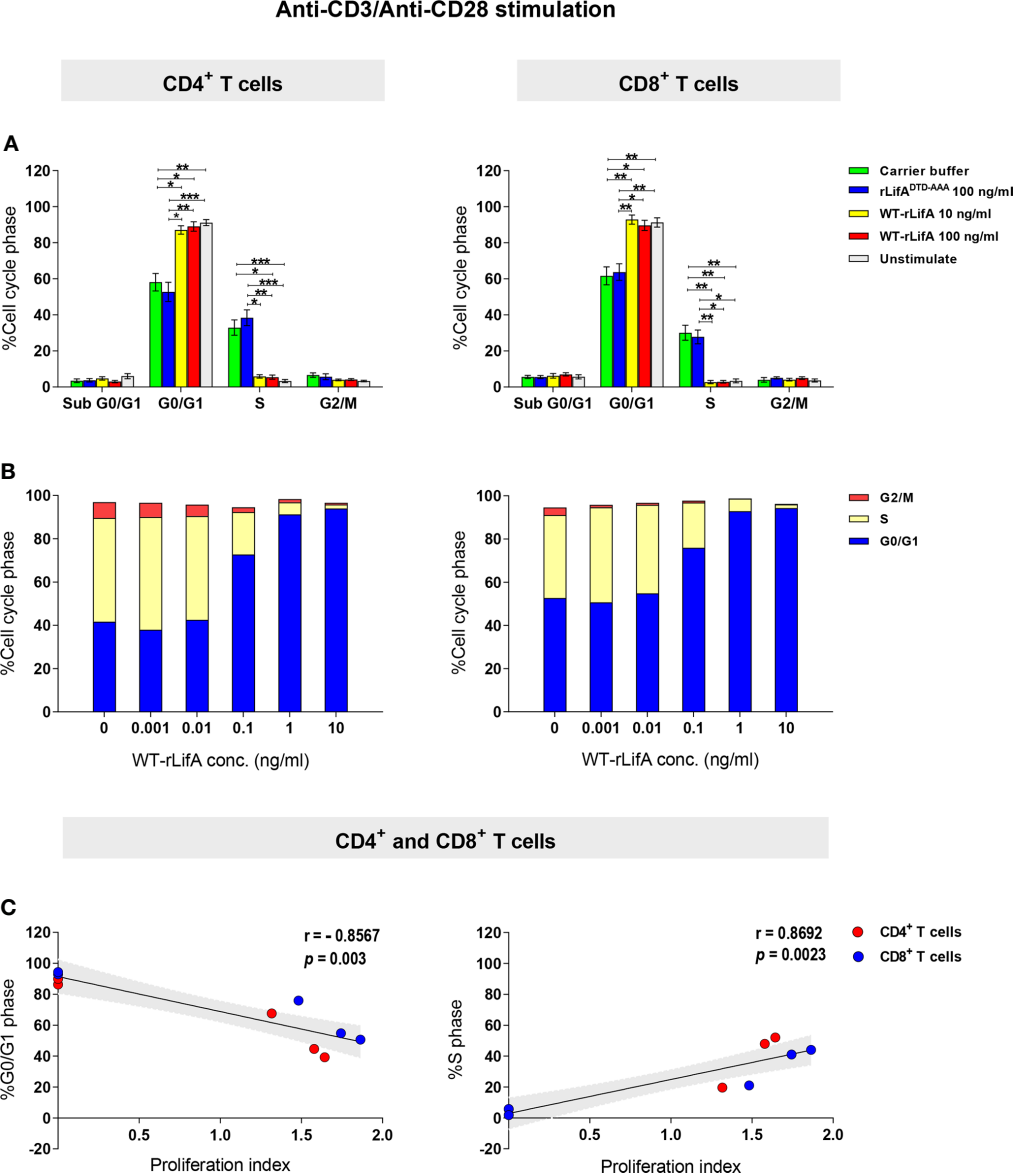
Lymphostatin arrests the cell cycle in G0/G1 phase in anti-CD3/anti-CD28-stimulated CD4^+^ and CD8^+^ T cells. **(A)** Distribution of sub G0/G1, G0/G1, S, and G2/M phases in the unstimulated (without treatment) and anti-CD3/anti-CD28-stimulated CD4^+^ and CD8^+^ T cells treated with rLifA^DTD-AAA^, WT-rLifA, or carrier buffer. Data represent the mean of 8 healthy donors ± S.E.M. *p < 0.05, **p < 0.01, ***p < 0.001 using Kruskal-Wallis test followed by Dunn’s test. **(B)** Distribution of G0/G1, S, and G2/M phases in anti-CD3/anti-CD28-stimulated CD4^+^ and CD8^+^ T cells treated with varied concentrations of WT-rLifA or left untreated (cells with mitogen; no tested protein). Data are from 3 healthy donors. **(C)** A Spearman’s rank-order correlation was run to determine the relationship between proliferation and cell cycle distribution of the percentage of cells in G0/G1 and S phases in anti-CD3/anti-CD28-stimulated CD4^+^ and CD8^+^ T cells treated with WT-rLifA (0.001 to 10 ng/ml). Each circle represents the mean of proliferation index from 8 healthy donors. The black line is the regression line; the shaded area is the 95% Confidence Interval.

Next, we repeated the analysis using CD4^+^ and CD8^+^ T cells from the blood of 8 healthy donors and quantified the proportions in different phases of the cell cycle. When stimulated with anti-CD3/anti-CD28 and treated with 10 and 100 ng/ml of WT-rLifA, CD4^+^ and CD8^+^ T cells from all donors predominantly accumulated in G0/G1 phase, as plotted in histograms (**Figure 10A**). The percentage of WT-rLifA-treated CD4^+^ and CD8^+^ cells in G0/G1 and S phases were significantly different compared to cells treated with buffer or rLifA^DTD-AAA^ (**Figure 10A**). The sub-G1 phase, representing apoptotic cells, was minimally detected in the presence of WT-rLifA at both 10 and 100 ng/ml and did not show any significant difference when compared to buffer and rLifA^DTD-AAA^-treated cells (**Figure 10A**). This finding correlates to the low numbers of apoptotic T cells detected by annexin V staining.

To examine whether lymphostatin affects the cell cycle in a concentration-dependent manner, the concentration of WT-rLifA was varied from 0.001 to 10 ng/ml using cells isolated from three independent healthy donors. For CD4^+^ and CD8^+^ T cells, the percentage of cells in G0/G1 phase increased with rising concentrations of WT-rLifA, while the percentage in S phase was correspondingly reduced (**Figure 10B**). We found a strong positive correlation between proliferation vs. the percentage of cells in S phase (*p* = 0.0023) but strong negative correlation between proliferation vs. the percentage of cells in G0/G1 phase (*p* = 0.003) in T cells treated with WT-rLifA at 0.001-10 ng/ml (**Figure 10C**).

Our data suggest that lymphostatin halts progression of anti-CD3/anti-CD28-stimulated human T cell subsets to S phase, leading to accumulation in the G0/G1 phase in a manner dependent on concentration and the DTD glycosyltransferase motif.

### Lymphostatin induces down-regulation of cyclins D3, E, A, up-regulation of p27, and prevents Rb phosphorylation in anti-CD3/anti-CD28-stimulated human T lymphocytes

In mammalian cells, cyclins and cdks play a critical role in regulating the cell cycle. Some of negative cell cycle regulators such as p53, pRb, p27, and p21 play an antagonistic role to halt cells from moving to subsequent cycle phases (Vermeulen et al., 2003). Briefly, pRb protein is a tumour suppressor and acts as a major restriction point in the G0/G1 phase. In the G0 and early G1 phase, pRb is associated with the E2F factor and retained in hypophosphorylated form. Cyclin D in association with cdk4 and cdk6 hyperphosphorylate pRb (ppRb) causing E2F to be released, allowing stimulation of genes necessary for G1/S progression, e.g. cyclin E (Kasten and Giordano, 1998; Sherr and Roberts, 1999). A cyclin-cdk inhibitor, p27^kip1^, has a function to maintain cells in the quiescent state by inactivating cyclin E-cdk2 to control cell cycle progression at G1 (Sherr and Roberts, 1999; Sun et al., 2016). To drive cycle progression, high levels of cyclin E titrate p27^kip1^ away from cyclin E-cdk2 heterodimers, allowing cyclin E-cdk2 to phosphorylate p27^kip1^ and finally its ubiquitin-mediated degradation (Montagnoli et al., 1999; Tsvetkov et al., 1999). Cyclin A levels peak in the S phase whereas high levels of cyclin B are present in the M phase (Vermeulen et al., 2003).

**Figure 11:**
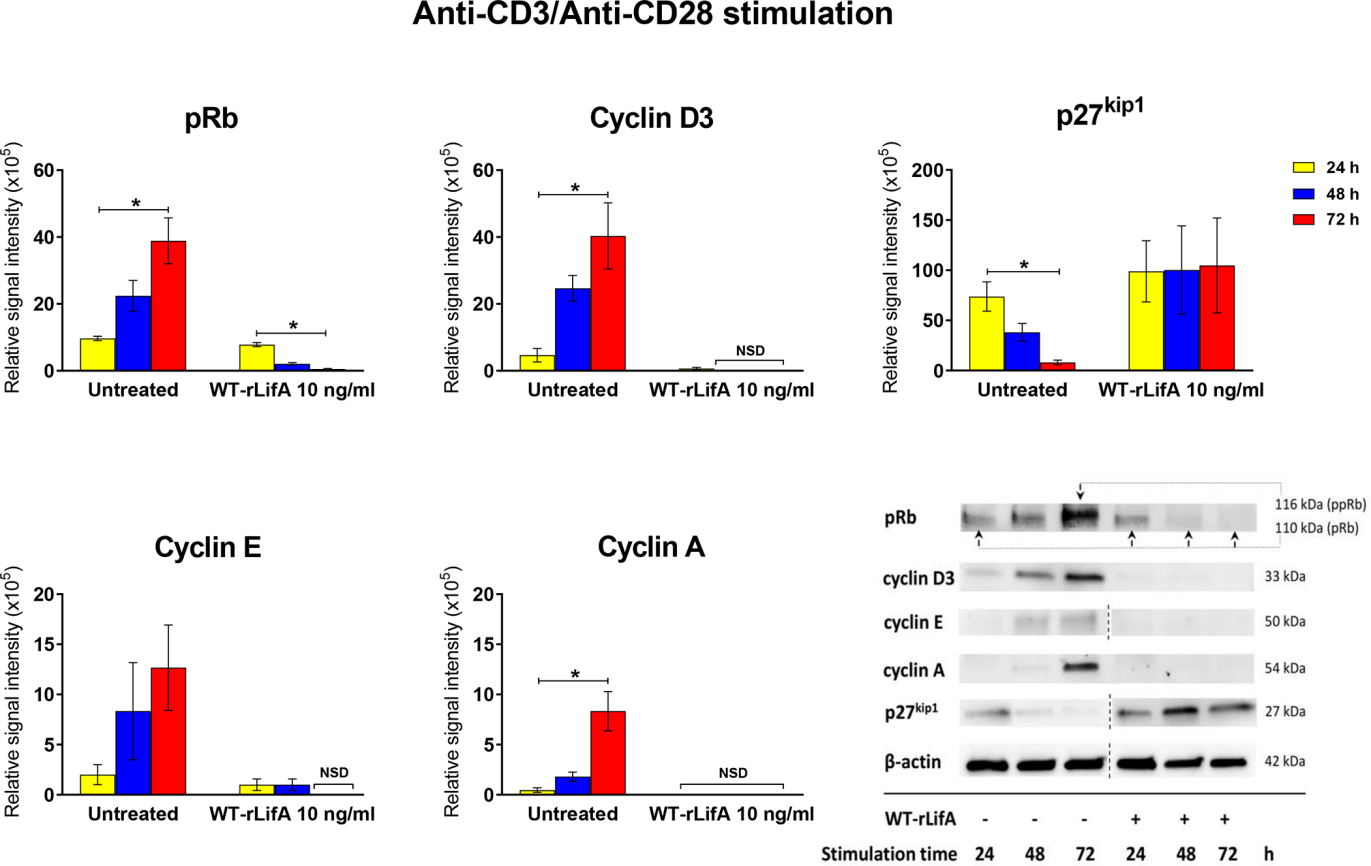
Lymphostatin inhibits expression of cell cyclins and pRb while causing accumulation of the cell cycle inhibitor p27^kip1^. Bar graphs showing densitometric analysis of cell cycle proteins (pRb, p27^kip1^, cyclins D3, E, and A) detected by Western blot in anti-CD3/anti-CD28-stimulated T cells treated with 10 ng/ml of WT-rLifA compared to untreated cells (cells with mitogen; no tested protein) at varied stimulation time. Data are derived from analysis of cells from 3 healthy donors. Data are expressed as mean ± S.E.M. *p < 0.05 using Kruskal-Wallis test followed by Dunn’s test. NSD means no signal detected. Representative immunoblot image showing the abundance of cell cycle proteins in anti-CD3/anti-CD28-stimulated T cells treated with 10 ng/ml of WT-rLifA compared to untreated cells at varied stimulation time. Data are representative of cells from one donor. The dotted line indicates that some non-relevant lanes were cropped to aid interpretation.

Based on evidence that lymphostatin causes T cells to accumulate in the G0/G1 phase, we investigated expression of key cyclins and cell-cycle regulating proteins mainly in the G1 to S cell cycle transition; specifically cyclins D3, E, and A, and the pRb and p27^kip1^ proteins. As shown in **Figure 11**, the levels of cyclins D3, E and A were markedly lower in WT-rLifA-treated T cells over time, whereas they increased in abundance from 24 to 72 h post-stimulation with anti-CD3/anti-CD28 in the absence of lymphostatin. Similarly, level of pRb declined over time in WT-rLifA-treated cells (**Figure 11**). Conversely, the relative levels of the p27^kip1^ protein were markedly increased in the WT-rLifA-treated T cells compared to untreated cells (**Figure 11**). Densitometric analysis across 3 independent donors normalized against β-actin as a loading control, revealed significant differences in the signal intensities of pRb, p27, cyclin D3, and cyclin A between 24 h and 72 h stimulation for untreated T cells (**Figure 11**). In the WT-rLifA treated T cells, a significant difference of the pRb signal intensity was found between 24 h and 72 h stimulation (**Figure 11**). The data are consistent with arrest of the cells in G0/G1 phase and inability to progress at the G1 to S checkpoint.

### Lymphostatin widely alters the phosphorylation of regulatory proteins in T cells following anti-CD3/Anti-CD28 stimulation

We next sought to determine if effects of lymphostatin on lymphocyte function could be explained by inhibition of signal transduction pathways that regulate key cellular functions. For this, we used a Proteome Profiler kit to study levels of phosphorylated proteins by antibody-mediated capture from lysates of T cells stimulated with anti-CD3/anti-CD28 and compared between WT-rLifA treated and untreated T cells.

**Figure 12:**
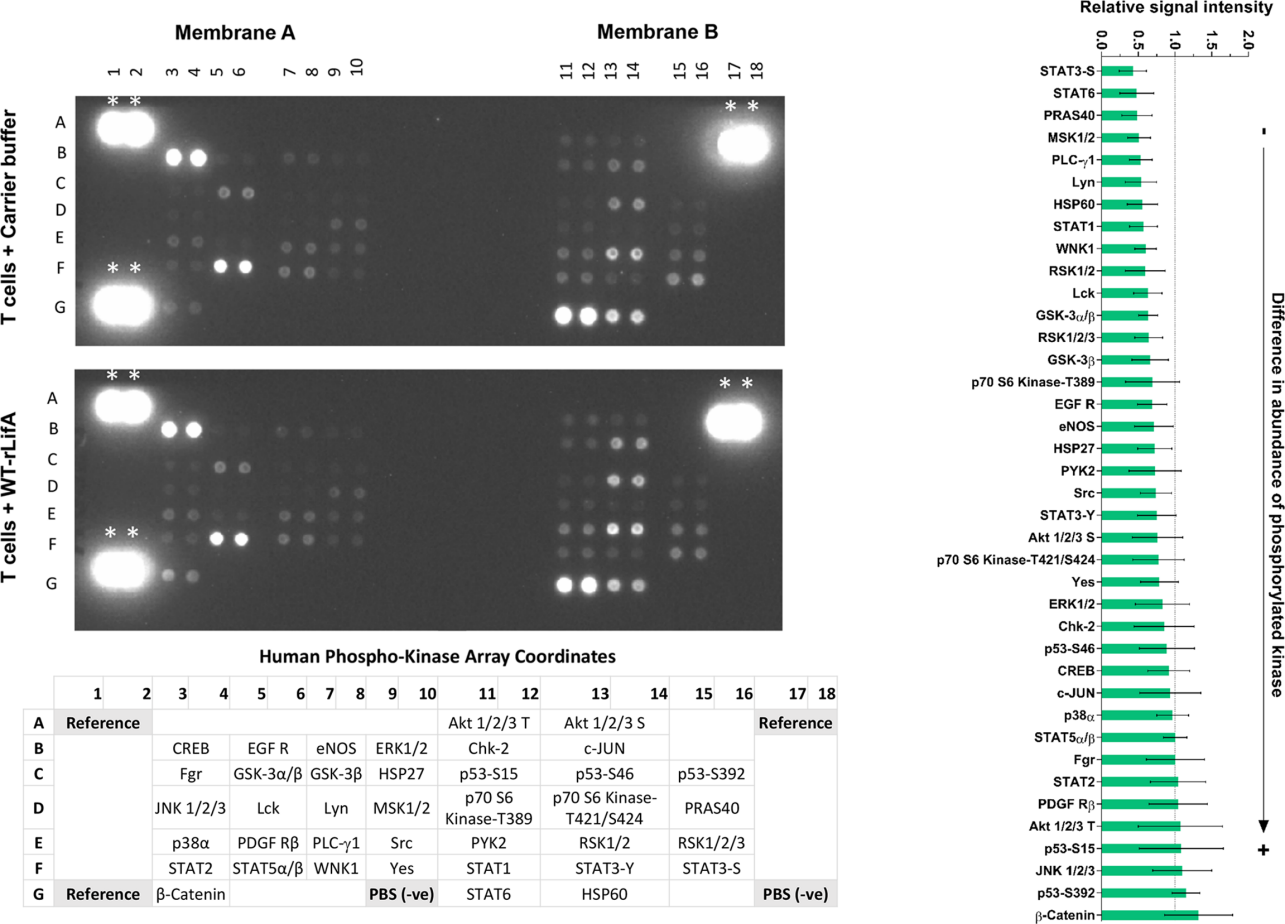
Lymphostatin broadly affects the phosphorylation of kinases involved in signal transduction in anti-CD3/anti-CD28-stimulated T cells. Left panel shows immunoblot of signaling phosphokinases in anti-CD3/anti-CD28-stimulated T cells treated with 10 ng/ml of WT-rLifA or untreated (cells with mitogen; no tested protein). Representative data using cells from one donor is shown. The array comprises immobilized antibodies specific for phosphorylated variants of the kinases shown at the bottom of membranes. *indicates as reference spots. Bar graph on the right panel shows relative signal intensity of phosphorylated kinases captured from ant-CD3/anti-CD28-stimulated T cells treated with 10 ng/ml of WT-rLifA. Data represent the mean of 5 independent donors ± S.E.M. Statistical comparison was performed by using Kruskal-Wallis test.

As shown in **Figure 12**, levels of 30 of a total 39 phosphorylated signaling proteins were decreased in the presence of 10 ng/ml WT-rLifA. **Table S1** showed that 30 of 39 proteins are kinases from key signaling pathways including members of the TCR (Ibiza et al., 2006; Rossy et al., 2012; Zeng et al., 2021), Janus kinase/signal transducers and activators of transcription (JAK/STAT) (Ng and Cantrell, 1997; O’Shea, 1997), P13K/Akt/mTOR (Wang et al., 2020), mitogen-activated protein kinase (MAPK)/extracellular-signal-regulated kinase (Erk) (Morrison, 2012), and p38-MAPK (Roux and Blenis, 2004) pathways. For the remaining 9 proteins studied, phosphorylation levels of STAT5α/β and Fgr remained unaltered whereas phosphorylation of STAT2, PDGF Rβ, threonine-phosphorylated Akt 1/2/3, p53-S15, JNK 1/2/3, p53-S392, and β-Catenin had moderately elevated levels as mean normalized intensity was ranged between 1.042 to 1.322.

Densitometric analysis of signal intensities for each protein across five independent donors indicated that none of the observed differences reached statistical significance. Consequently, and in the absence of validation of differences with specific antibodies, the impact of lymphostatin on specific kinases or pathways cannot be inferred with confidence. It is evident however, that lymphostatin treatment suppresses phosphorylation of a wide range of signaling proteins, many of which are downstream of TCR activation.

## Discussion

Studies thus far on the effect of lymphostatin on human cells have mostly relied on crude bacterial lysates and bulk PBMCs stimulated with mitogens (Klapproth et al., 1995; Klapproth et al., 1996). While Cassady-Cain and colleagues (Cassady-Cain et al., 2017) reported inhibition across bovine T cell subsets using highly purified lymphostatin, it was not clear if this applied to human T cells. We observed concentration-dependent inhibition of proliferation of human CD4^+^ and CD8^+^ T cells by lymphostatin when induced *via* TCR cross-linking by anti-CD3/anti-CD28 stimulation, but not when stimulated by PMA/ionomycin in a way that bypasses the TCR. The N-terminal DTD glycosyltransferase motif was necessary for inhibitory activity against human T cells, consistent with data using bovine T cells (Cassady-Cain et al., 2016).

The ED_50_ of WT-rLifA was in the femtomolar range against human T cells (364 fM and 342 fM for CD4^+^ and CD8^+^, respectively). These levels are c. 2.5-fold greater than reported against bovine T cells (138 fM) (Cassady-Cain et al., 2017). This could be due to species-specific differences in sensitivity of the cells, variation between batches of purified protein or the use of different strategies to quantify cell proliferation. Proliferation of bovine T lymphocytes was measured by analysis of cellular metabolic activity (Cassady-Cain et al., 2017), whereas we used CFSE flow cytometry to measure proliferation at a single cell level. This offers a number of advantages over simpler methods for assessing proliferation (e.g. thymidine incorporation), as we could directly visualize proliferating T cell subsets in the CFSE-labeled PBMCs and multi-fluorochrome staining allowed us to detect CD4^+^ and CD8^+^ T cell populations when using PBMCs. In addition, flow cytometry of cells subject to CFSE and live/dead staining allowed us to monitor proliferative responses in parallel with cytotoxicity within the same T cell populations. Our data indicate that the WT-rLifA-treated CD4^+^ and CD8^+^ T cells remain viable at concentrations of LifA sufficient to block cell proliferation. Thus lymphostatin is not directly cytotoxic, consistent with data from lactate dehydrogenase release assays using bovine T cells (Cassady-Cain et al., 2016). Further, unlike some bacterial virulence factors that interfere with lymphocyte function by inducing apoptosis (reviewed in Cassady-Cain et al., 2018), we showed that lymphostatin did not induce apoptosis in anti-CD3/anti-CD28-stimulated CD4^+^ and CD8^+^ T cells at concentrations at which their proliferation was inhibited.

We also demonstrated that lymphostatin inhibited major cytokines produced by CD4^+^ and CD8^+^ T cells following TCR stimulation. Further, it could be implied that lymphostatin inhibited cytokine synthesis of Th1, Th2, and Treg subpopulations as production of signature cytokines from those CD4^+^ T cell subsets was found to be suppressed (IL-2 and IFN-γ for Th1, IL-4 for Th2, and IL-10 for Treg). These results of cytokine inhibition fully concur with the previous observations using human PBMCs (Klapproth et al., 2000) and isolated bovine T cells (Cassady-Cain et al., 2017). Cytokine responses were also consistent with the lack of inhibition of T cell proliferation stimulated by PMA/ionomycin, suggesting that lymphostatin does not affect cytokine synthesis of CD4^+^ and CD8^+^ T cells following such stimulation. Additionally, we show for the first time that the DTD motif of lymphostatin is required for inhibition of cytokine production in human CD4^+^ and CD8^+^ T cells following TCR stimulation. Further, we report that lymphostatin appears to act early after TCR activation, as down-regulation of CD69 was detected in CD4^+^ and CD8^+^ T cells treated with WT-rLifA in a concentration-dependent manner. The concentration that suppressed CD69 expression profoundly diminished cytokines produced by CD4^+^ and CD8^+^ T cells. Further studies are required to explore the extent to which lymphostatin can inhibit lymphocyte proliferation and cytokine synthesis in the gut mucosa and peripheral circulation in animal models, and the extent to which this impacts the development of antigen-specific adaptive responses.

Some enteric bacterial pathogens produce proteins that interfere with progression of the cell cycle (El-Aouar Filho et al., 2017). These so-called cyclomodulins typically impede cell division by acting on regulators of the cell cycle and are believed to prolong colonization within the host (El-Aouar Filho et al., 2017). In EPEC, a Type 3 secreted effector termed the cycle inhibiting factor (CIF) acts as a cyclomodulin by regulating progression at both the G1/S and G2/M transitions as shown high accumulation of cdk inhibitors p21^Cip1^ and p27^Kip1^ (Marches et al., 2003; Samba-Louaka et al., 2008; Morikawa et al., 2010; Taieb et al., 2011). In our study, we found compelling evidence, using cells from 8 healthy donors, for high accumulation of lymphostatin-treated cells in the G0/G1 phase following anti-CD3/anti-CD28-stimulated T cells. Arrest in the G0/G1 phase was strongly dependent on the N-terminal DTD motif and, assuming lymphostatin acts as a glycosyltransferase, further studies will be needed to define the cellular target(s) of glycosylation and how this impacts on the cell cycle.

Gerhard and colleagues (Gerhard et al., 2005) reported a secreted low molecular weight factor from *H. pylori* caused human T cells treated with phytohaemagglutinin (which cross-links the TCR and multiple cell surface glycoproteins) to accumulate in G1 phase. The factor interfered with expression of cyclin D3, cyclin E, cyclin A, pRb and p27^Kip1^ proteins. It has been reported that *H. pylori* VacA strongly impaired IL-2-induced cell cycle progression in anti-CD3/anti-CD28-activated human T cells by causing arrest in the G1 phase (Sundrud et al., 2004). Another virulence factor of *H. pylori*, gamma-glutamyl transpeptidase (GGT), has been reported to cause G1 cell cycle arrest in Jurkat T cells, as characterized by increasing levels of cdk inhibitor p27^Kip1^ but profoundly reduced levels of cyclin D3 and cyclin E1 (Schmees et al., 2007). It is possible therefore that enteric pathogens have evolved convergent strategies to interfere with lymphocyte function and the extent to which the underlying mechanisms are common requires further study. Our data showed that analysis of the abundance in phosphorylation of cyclin D3, cyclin E, cyclin A, cdk inhibitor p27^Kip1^, and major G1 checkpoint pRb are consistent with lymphostatin causing G0/G1 phase arrest and suppressing entry to S phase. The distribution of cells in S and G0/G1 phases was strongly associated with lymphostatin concentration. To the best of our knowledge, this is the first evidence of such activity and it will be of interest to determine if such arrest occurs with intraepithelial or peripheral lymphocytes *in vivo*.

IL-2 is an important factor to eliminate cdk inhibitor p27^Kip1^, aiding transition from quiescence to S phase in T lymphocytes (Nourse et al., 1994). Moreover, IL-2 is a key regulator of T cell responses and secreted IL-2 acts as an extrinsic factor to promote T cell survival (Linsley and Ledbetter, 1993; Ross and Cantrell, 2018). Our data showed that p27^Kip1^ was strongly accumulated in human T cells treated with LifA at 10 ng/ml, while at 10-fold lower concentration (1 ng/ml) lymphostatin fully inhibited IL-2 production. While it is tempting to speculate that the lack of IL-2 may influence accumulation of p27^Kip1^ and thus explain arrest of the cell cycle and absence of proliferation, it should be noted that lymphostatin can inhibit IL-2 stimulated proliferation of bovine T cells (Cassady-Cain et al., 2017). Nonetheless, merit exists in establishing if arrest of lymphostatin-treated human T cells in G0/G1 phase still occurs in the presence of exogenous recombinant IL-2.

In this study, we also determined the impact of lymphostatin on the abundance of phosphorylated kinases involved in cell signaling. While no significant differences were detected upon lymphostatin treatment of T cells from five separate donors, there were reductions in the abundance of 30 out of 39 of the phosphokinases, further studies are needed to validate the nature and magnitude of differences with specific antibodies and determine if lymphostatin can still act in the presence of inhibitors of specific pathways.

Taken together, our data provide valuable novel insights into the mode of action of lymphostatin against human T cells. It will be fascinating to explore the extent to which this activity explains the key role the protein plays in intestinal colonization by attaching & effacing *E. coli* and whether these pathogens employ lymphostatin to suppress intestinal gut immune responses e.g. gut innate γδ T cells, adaptive T cells, and antibody responses.

## Data availability statement

The raw data supporting the conclusions of this article will be made available by the authors, without undue reservation.

## Ethics statement

The studies involving human participants were reviewed and approved by the Siriraj Institutional Review Board (SIRB) committee, Faculty of Medicine Siriraj Hospital, Mahidol University, under approval no. 724/2562(IRB2). The patients/ participants provided their written informed consent to participate in this study.

## Author contributions

SK and MS conceived and designed the experiments; DR, JS, and KP provided consultation for method development; SJ arranged study materials; NR performed the experiments, generated and analysed the data; NR and MS wrote the manuscript; DR, SP, KP, SD, MS, and SK critically reviewed and edited the manuscript. All authors read and approved the final version of the manuscript.

## Funding

This work was supported by grants from the Biotechnology & Biological Sciences Research Council (Institute Strategic Programme Grant BBS/E/D/20002173) and the Siriraj Research Fund, Grant Number (IO) R016433012, Faculty of Medicine Siriraj Hospital, Mahidol University.

## Acknowledgments

We would like to thank Dr. Methichit Wattanapanitch at the Siriraj Center for Regenerative Medicine (SiCRM), Research Department, Faculty of Medicine Siriraj Hospital, Mahidol University for providing the Jurkat T cell line. We are thankful Mr. Kosol Yongvanitchit at the Department of Bacterial and Parasitic Diseases, AFRIMS for kind assistance in flow cytometry assay and data analysis.

Disclaimer: Material has been reviewed by the Walter Reed Army Institute of Research. There is no objection to its presentation and/or publication. The opinions or assertions contained herein are the private views of the author, and are not to be construed as official, or as reflecting true views of the Department of the Army or the Department of Defense. The investigators have adhered to the policies for protection of human subjects as prescribed in AR 70–25.

## Conflict of interest

The authors declare that the research was conducted in the absence of any commercial or financial relationships that could be construed as a potential conflict of interest.

## Publisher’s note

All claims expressed in this article are solely those of the authors and do not necessarily represent those of their affiliated organizations, or those of the publisher, the editors and the reviewers. Any product that may be evaluated in this article, or claim that may be made by its manufacturer, is not guaranteed or endorsed by the publisher.

## Supplementary material

Supplementary Figure 1 SDS-PAGE and Western blot analysis of the purified rLifA proteins.

Supplementary Table 1 Phosphokinases and their functions in cellular signaling.
