# Supplementary material for: Lymphostatin, a virulence factor of attaching and effacing Escherichia coli, inhibits proliferation and cytokine responses of human T cells in a manner associated with cell cycle arrest but not apoptosis or necrosis
Source: Front Cell Infect Microbiol. 2022 Jul 29;12:941939. doi: 10.3389/fcimb.2022.941939 (PMC9373022; doi:10.3389/fcimb.2022.941939)
Supplement: Supplementary Figure 1 — SDS-PAGE and Western blot analysis of the purified rLifA proteins. [file DataSheet_1.docx]

Supplementary Material

# Table S1 Phosphokinases and their functions in cellular signaling.

| **Phosphokinases detected at lower abundance in WT-rLifA-treated T cells** | **Description** | **References** |
| --- | --- | --- |
| Lck, Src, PLCγ1, eNOS | TCR signaling | Ibiza et al., 2006; Rossy et al., 2012; Zeng et al., 2021 |
| STAT1, STAT3, STAT6 | JAK/STAT   - STAT1; IFN-γ signaling - STAT3; Targets for multiple kinase pathways in T cells - STAT6; Th2 differentiation | Ng and Cantrell, 1997; O'Shea, 1997 |
| PRAS40, Akt 1/2/3 S | P13K/Akt/mTOR signaling   - PRAS40 is substrate for Akt and mTORC | Wang et al., 2020 |
| ERK1/2, RSK1/2/3, p70 S6 | MAPK/ERK signaling | Morrison, 2012 |
| MSK1/2, HSP27 | ERK and p38-MAPK signaling | Roux and Blenis, 2004 |
| EGF-R | EGFR signaling   - Involved in G1/S cell cycle progression | Wee and Wang, 2017 |
| Lyn | B cell receptor signaling | Gauld and Cambier, 2004 |
| HSP60 | TLR2-signaling signaling | Quintana et al., 2008 |
| Wnk1 | WNK signaling   - Regulates T cell adhesion and migration | Kochl et al., 2016 |
| GSK-3αβ | Regulates PD-1 expression | Rudd et al., 2020 |
| chk2 | Chk signaling   - Involved in DNA damage checkpoints | Shaltiel et al., 2015 |
| p53 S46 | p53 signaling   - Promotes apoptosis and/or cell cycle arrest | Liu et al., 2019 |

**
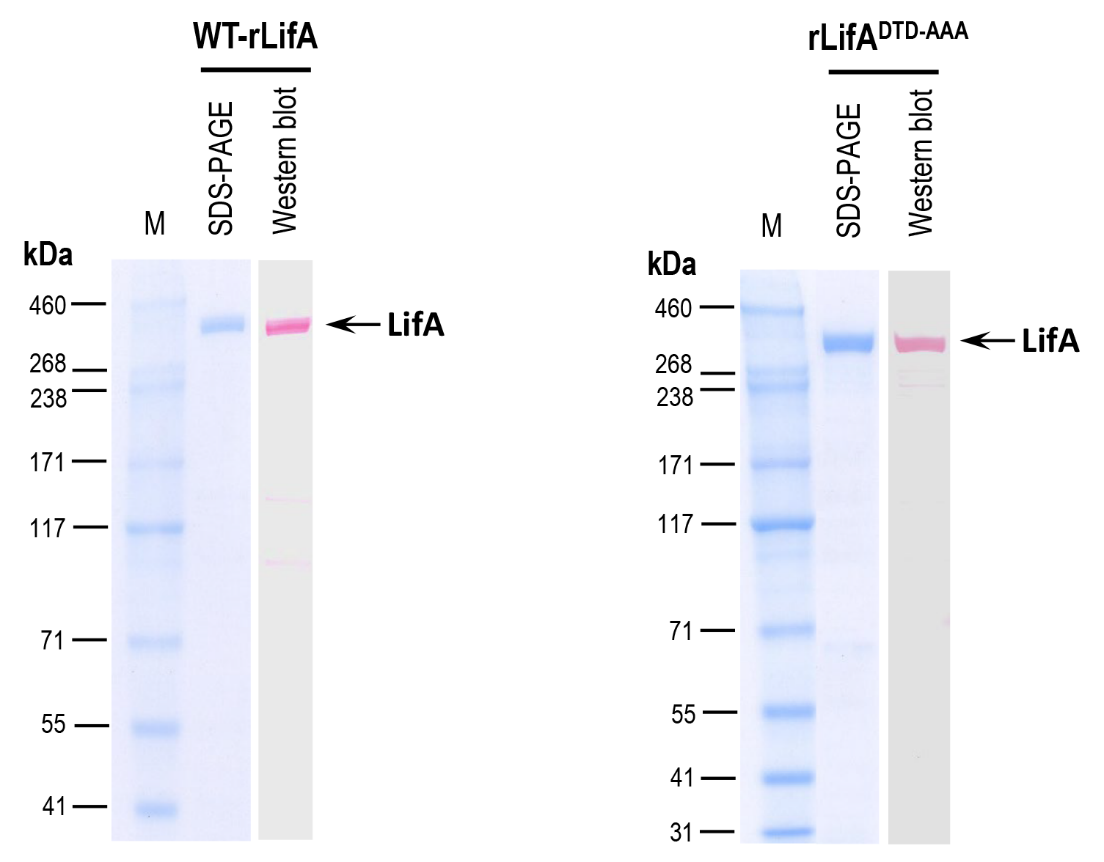
**

**Figure S1** SDS-PAGE and western blot analysis of the purified rLifA proteins. A total of 1 μg of purified protein (left panel; WT-rLifA and right panel; rLifA^DTD-AAA^) was subjected to 3 - 8% Tris-Acetate protein gel for SDS-PAGE. Then the protein gels were electro-transferred to nitrocellulose membrane for western blot analysis. The arrows indicate molecular weight of approximately 365 kDa for the purified WT-rLifA and rLifA^DTD-AAA^ proteins. M indicates HiMark Pre-stained Protein Standards.

**References**

Gauld, S.B., and Cambier, J.C. (2004). Src-family kinases in B-cell development and signaling. *Oncogene.* 23, 8001-8006. doi: 10.1038/sj.onc.1208075

Ibiza, S., Victor, V.M., Bosca, I., Ortega, A., Urzainqui, A., O'Connor, J.E., et al. (2006). Endothelial nitric oxide synthase regulates T cell receptor signaling at the immunological synapse. *Immunity.* 24, 753-765. doi: 10.1016/j.immuni.2006.04.006

Kochl, R., Thelen, F., Vanes, L., Brazao, T.F., Fountain, K., Xie, J., et al. (2016). WNK1 kinase balances T cell adhesion versus migration *in vivo*. *Nat. Immunol.* 17, 1075-1083. doi: 10.1038/ni.3495

Liu, Y., Tavana, O., and Gu, W. (2019). p53 modifications: exquisite decorations of the powerful guardian. *J. Mol. Cell. Biol*. 11, 564-577. doi: 10.1093/jmcb/mjz060

Morrison, D.K. (2012). MAP kinase pathways. Cold. Spring. Harb. Perspect. Biol. 4. doi: 10.1101/cshperspect.a011254

Ng, J., and Cantrell, D. (1997). STAT3 is a serine kinase target in T lymphocytes. Interleukin 2 and T cell antigen receptor signals converge upon serine 727. *J. Biol. Chem*. 272, 24542-24549. doi: 10.1074/jbc.272.39.24542

O'Shea, J.J. (1997). Jaks, STATs, cytokine signal transduction, and immunoregulation: are we there yet? *Immunity.* 7, 1-11. doi: 10.1016/s1074-7613(00)80505-1

Quintana, F.J., Mimran, A., Carmi, P., Mor, F., and Cohen, I.R. (2008). HSP60 as a target of anti-ergotypic regulatory T cells. *PLoS ONE*. 3, e4026. doi: 10.1371/journal.pone.0004026

Rossy, J., Williamson, D.J., and Gaus, K. (2012). How does the kinase Lck phosphorylate the T cell receptor? spatial organization as a regulatory mechanism. *Front. Immunol*. 3, 167. doi: 10.3389/fimmu.2012.00167

Roux, P.P., and Blenis, J. (2004). ERK and p38 MAPK-activated protein kinases: a family of protein kinases with diverse biological functions. *Microbiol. Mol. Biol*. Rev. 68, 320-344. doi: 10.1128/MMBR.68.2.320-344.2004

Rudd, C.E., Chanthong, K., and Taylor, A. (2020). Small molecule inhibition of GSK-3 specifically inhibits the transcription of inhibitory co-receptor LAG-3 for enhanced anti-tumor immunity. *Cell. Rep*. 30, 2075-2082 e2074. doi: 10.1016/j.celrep.2020.01.076

Shaltiel, I.A., Krenning, L., Bruinsma, W., and Medema, R.H. (2015). The same, only different - DNA damage checkpoints and their reversal throughout the cell cycle. *J. Cell. Sci*. 128, 607-620. doi: 10.1242/jcs.163766

Wang, P., Zhang, Q., Tan, L., Xu, Y., Xie, X., and Zhao, Y. (2020). The regulatory effects of mTOR complexes in the differentiation and function of CD4(+) T cell subsets. *J. Immunol. Res*. 2020, 3406032. doi: 10.1155/2020/3406032

Wee, P., and Wang, Z. (2017). Epidermal growth factor receptor cell proliferation signaling pathways. *Cancers (Basel).* 9. doi: 10.3390/cancers9050052

Zeng, L., Palaia, I., Saric, A., and Su, X. (2021). PLCγ1 promotes phase separation of T cell signaling components. *J. Cell. Biol*. 220, e202009154. doi: 10.1083/jcb.202009154
